# Strategies and cognitive reserve to preserve lexical production in aging

**DOI:** 10.1007/s11357-021-00367-5

**Published:** 2021-05-10

**Authors:** Monica Baciu, Sonja Banjac, Elise Roger, Célise Haldin, Marcela Perrone-Bertolotti, Hélène Lœvenbruck, Jean-François Démonet

**Affiliations:** 1 Univ. Grenoble Alpes, CNRS LPNC UMR 5105, 38000 Grenoble, France; 2 Leenaards Memory Centre, University Hospital of Lausanne, Lausanne, Switzerland

**Keywords:** Healthy aging, Lexical, Production, Behavioral, Brain, Reserve factors

## Abstract

In the absence of any neuropsychiatric condition, older adults may show declining performance in several cognitive processes and among them, in retrieving and producing words, reflected in slower responses and even reduced accuracy compared to younger adults. To overcome this difficulty, healthy older adults implement compensatory strategies, which are the focus of this paper. We provide a review of mainstream findings on deficient mechanisms and possible neurocognitive strategies used by older adults to overcome the deleterious effects of age on lexical production. Moreover, we present findings on genetic and lifestyle factors that might either be protective or risk factors of cognitive impairment in advanced age. We propose that “aging-modulating factors” (AMF) can be modified, offering prevention opportunities against aging effects. Based on our review and this proposition, we introduce an integrative neurocognitive model of mechanisms and compensatory strategies for lexical production in older adults (entitled Lexical Access and Retrieval in Aging, LARA). The main hypothesis defended in LARA is that cognitive aging evolves heterogeneously and involves complementary domain-general and domain-specific mechanisms, with substantial inter-individual variability, reflected at behavioral, cognitive, and brain levels. Furthermore, we argue that the ability to compensate for the effect of cognitive aging depends on the amount of reserve specific to each individual which is, in turn, modulated by the AMF. Our conclusion is that a variety of mechanisms and compensatory strategies coexist in the same individual to oppose the effect of age. The role of reserve is pivotal for a successful coping with age-related changes and future research should continue to explore the modulating role of AMF.

**Supplementary Information:**

The online version contains supplementary material available at 10.1007/s11357-021-00367-5.

## Cognitive aging: a focus on lexical production

As average lifespan increases, the proportion of older people is growing exponentially across the globe. In 2050, people over the age of 65 are expected to make 16.7% of the world’s population ([78]; cited by [87]) and the number of individuals over 60 is projected to triple [8]. This will result in increased demands in terms of health care costs for an aging population, together with increased family, societal, and economic burden. In order to promote healthy aging and prevent neurodegenerative disorders, it is crucial to better understand the adverse processes associated with aging. Cognitive aging is not a uniform process, as some cognitive abilities are significantly impaired by age, while others remain unaffected or can even improve. Decreases in the speed of information processing [103], decrements of encoding in episodic memory ([99, 112]; see also [11] for a review), and dysfunctions of working memory [107, 137] are generally observed in older adults. Fechner et al. [55] argued that aging affects “the flexibility to form, to switch, and update representations in working memory.” Executive functions have an important role, as a “key contributor to age-related declines in a range of cognitive tasks” [66], with a decline in decision-making [55, 74], mental flexibility and inhibitory processes [33, 193], and attention [64, 106]. Conversely, over-learned skills and tasks based on individual experience and semantic knowledge may remain unaffected for longer or can even improve with age. Indeed, speech and language skills appear to remain more stable in healthy older adults (see [106], cited by [128]), as well as semantic memory [134], verbal comprehension [179], and vocabulary [73, 138].

Despite overall language skills preservation, some linguistic operations can be impaired by age [68, 126, 144]. For instance, verbal fluency, word retrieval, or confrontation naming can be progressively altered even if older adults may show greater lexical knowledge compared to younger adults [157, 199] due to longer accumulated linguistic experience and greater resistance of semantic memory [23]. It is admitted that language comprehension is more resistant to age than language production (see [27, 43, 149, 162, 166, 207]), a discrepancy explained, according to Sheldon et al. [170], by the fact that older adults benefit from the semantic context or the increase of accumulated verbal knowledge through the lifespan [40]. In contrast, language production and specifically lexical generation which involves word retrieval and word production are more frequently impaired by age. Older adults have difficulties in retrieving names [35, 51, 88, 133] and encounter more tip-of-the-tongue situations [26, 34, 53, 135, 167], i.e., the feeling of knowing the meaning of a word without being able to find and produce the word form immediately. These difficulties are more frequent in adults above 70 years [198] but may also occur earlier [35, 191]. In the present paper, we will specifically focus on lexical production to understand the mechanisms underlying the effect of age on language skills and how some lexical production/cognitive strategies could contribute to healthy aging.

We remind that difficulties encountered by older people to produce words may have several origins. Lexical production involves many coordinated cognitive and linguistic processes which depend on a large brain network including interconnected regions within and between hemispheres and which have intricate time courses [165]. According to dominant neurocognitive models of lexical retrieval reviewed by Friedmann et al. [61], producing a word starts with accessing the conceptual system to retrieve and select the target conceptual representation, which is still not verbally formulated but includes semantic properties, and presumably visual as well as functional representations. Conceptual processing depends on a large set of regions including inferior parietal, middle temporal, fusiform, parahippocampal, dorso-medial, and ventro-medial prefrontal cortices, as well as posterior cingulate, anterior temporal, and anterior part of the inferior frontal gyrus (see also [85, 86, 141]). The activation of lexico-semantic and syntactic representations comes next, recruiting superior middle temporal gyrus, precuneus, inferior frontal, and prefrontal regions [85, 171]. The generated semantic-syntactic representation includes information about the meaning of a word, its semantic properties, and its syntactic function. It activates the phonological representation in the phonological lexicon, which includes metrical information (stress pattern, length) as well as segmental information (consonants, vowels). Phonological encoding has been shown to involve posterior parts of the left superior and middle temporal gyri (e.g., [61]). Some researchers argue for the additional recruitment of the posterior superior temporal gyrus and the temporo-parietal junction, but this remains debated [61, 71]. According to the set of models reviewed by Friedmann et al. [61], the output of the phonological encoding is transferred to the phonological buffer, which stores phonological representation until the word is fully uttered and which composes metrically and morphologically complex words from their morphemes. The phonological output buffer has itself been associated with an area within the Sylvian fissure at the parietal-temporal boundary called Spt [80]. As synthesized in Friedmann et al. [61], phonetic encoding is the stage during which the phonemic string is prepared for articulation and sent to the motor system. Although this is still a matter of debates, research on speech motor control has suggested that phonetic encoding (sometimes also called articulatory planning, e.g., [71]) includes two phases, motor planning and motor programming, that may dissociate in developmental or acquired speech disorders [102, 188, 196]. The regions involved in phonetic encoding include the inferior frontal, ventral premotor and motor cortices, supplementary motor area, and the anterior part of the insula, the thalamus, and the cerebellum [71, 85]. Overall, lexical production involves a vast network of interconnected nodes, linked with the semantic system for word meaning representations, with phonological and orthographic systems for word sound and spelling representations, with the motor system for articulatory representations, and with cognitive control systems, for monitoring (see [41, 61, 111, 115]).

Given the large number of operations that are needed for an adequate lexical production, its decline with age may be explained by different mechanisms, either language-specific (LS) or domain-general (DG). An exhaustive presentation of studies on this topic in recent years is beyond the scope of the present paper. We simply provide a synthesis of significant findings published on lexical retrieval and production in normal aging in Table 1. To do that, a literature search was conducted using the PubMed database during November 2020 for a systematic literature review of age-related lexical changes in lexical production, mainly based on a behavioral and neuropsychological approach. Keywords used were “aging/ageing”, “lexical”, “production”, “naming”, “tip-of-the-tongue”, “language”, “healthy” in different combinations (aging OR ageing) AND lexical AND production; (aging OR ageing) AND tip-of-the-tongue; (aging OR ageing) AND naming AND language). Two of the authors first screened the titles and abstracts of the resulting papers to assess their eligibility and then performed full-text scans to determine whether papers met the inclusion criteria. To be included in the review, the studies had to fulfill the following inclusion criteria: published between 2000 and 2020, written in English, the study includes older participants (60+), participants did not suffer from any form of pathology, tasks included one-word production, the study specifically assesses the relationship between lexical production and aging, and employed tasks focus explicitly on language. The studies were left out if they fulfilled one of the following exclusion criteria: tasks included written production, tasks included a sentence or narrative production, and only a neuroimaging approach. Case studies, meta-analysis, and review papers were excluded. Through this process, 46 papers were identified.

**Table 1:** Synthetic presentation of main studies reporting results on lexical retrieval and generation in normal aging. All information on the inclusion of studies is presented in the main text

Reference article	Method(s)	Participants	Tasks and tests	Analyses	Main results	LARA-based mechanisms
Moberg, M., Ferraro, F. R., & Petros, T. V. (2000). Lexical properties of the Boston Naming Test stimuli: age differences in word naming and lexical decision latency. Applied neuropsychology https://pubmed.ncbi.nlm.nih.gov/11125708/	Behavioral Neuropsychology	English-speakers; Experiment 1: 23 younger 19–36y; 25 older 60–91y; Experiment 2: 23 younger 18–36y, 31 older 60–75y	Wechsler Adult Intelligence Scale–Revised (WAIS–R), vocabulary test, Geriatric Depression Scale–Short Form (GDS–SF), Boston Naming Test (BNT), lexical decision	Correlation, multiple regression analysis	Lexical access not affected by age. Lexical decision performance predicted by age and increased latencies with age.	LS-lifespan
Evrard M. (2002). Ageing and lexical access to common and proper names in picture naming. Brain Language https://pubmed.ncbi.nlm.nih.gov/12081390/	Behavioral Neuropsychology	French-speakers; 35 aged 18–34y (14 males); 30 aged 35–54 (13 males); 33 aged 55–75 (16 males)	Binois and Pichot vocabulary test, Digit-Symbol subtest of the WAIS-R, picture naming task (objects and famous faces)	Analyses of variance (ANOVA)	Older experienced more tip-of-the-tongue states (TOTs) for proper names, but not for common names.	LS-lifespan LS-reserve
Taylor, J. K., & Burke, D. M. (2002). Asymmetric aging effects on semantic and phonological processes: naming in the picture-word interference task. Psychology and aging https://pubmed.ncbi.nlm.nih.gov/12507362/	Behavioral Neuropsychology	English-speakers; Experiment 1: 48 young aged 18–29, 48 older aged 62–85; Experiment 2: 32 young aged 18–22, 32 older aged 60–89.	Shipley Vocabulary, picture naming interference test	ANOVA	Asymmetric aging effects on semantic and phonological language processes: no evidence for age differences in bottom-up priming from phonologically related distractors. Distractors semantically related to the picture interfered more in older than in younger. Age-related deficit for top-down phonological priming. Aging enriches semantics.	LS-lifespan LS-reserve
Mackay, A. I., Connor, L. T., Albert, M. L., & Obler, L. K. (2002). Noun and verb retrieval in healthy aging. Journal of the International Neuropsychological Society : JINS https://pubmed.ncbi.nlm.nih.gov/12240740/	Behavioral Neuropsychology	English-speakers, 43 aged 50–59 (20 males), 63 aged 60–69 (33 males), 65 aged 70–79 (38 males)	Boston Naming Test, Action Naming Test, MMSE	Analysis of covariance (ANCOVA)	Retrieval of nouns and verbs declines equally with age. Participants older than 70 benefit more from phonemic cuing than from any other cue type for both nouns and verbs, suggesting common strategic mechanism for noun and verb retrieval. Reduced transmission of activation between lexical and phonological nodes.	LS-reserve
Morrison, C. M., Hirsh, K. W., & Duggan, G. B. (2003). Age of acquisition, ageing, and verb production: normative and experimental data. The Quarterly journal of experimental psychology. Human experimental psychology https://pubmed.ncbi.nlm.nih.gov/12745837/	Behavioral	English-speakers; 44 young aged 18–27, 30 older aged 65–87	Picture naming task (action verbs)	Multiple regression, ANOVA	Age of acquisition predicts naming speed in young and old adults, early-acquired verbs being more rapidly named than later-acquired verbs. Older are less accurate for naming and respond slower. Word frequency predicts picture naming speed only in older adults. Picture naming in older adults affected by the age of acquisition, lexical frequency, and other perceptive specificities.	DG-reserve LS-lifespan
Tsang, H. L., & Lee, T. M. (2003). The effect of ageing on confrontational naming ability. Archives of clinical neuropsychology: the official journal of the National Academy of Neuropsychologists https://pubmed.ncbi.nlm.nih.gov/14591480/	Behavioral	Cantonese Chinese-speakers, 30 young aged 14–22 (12 males), 30 old aged 60–86 (15 males)	Chinese Naming Test	ANCOVA, correlations	Younger show better naming performance than older (accuracy and response latency), unexplained by the level of education. Higher heterogeneity in terms of performance among older than younger subsamples suggesting variability of evolution with age.	DG-reserve LS-lifespan LS-reserve
Connor, L. T., Spiro, A., 3rd, Obler, L. K., & Albert, M. L. (2004). Change in object naming ability during adulthood. The journals of gerontology. Series B, Psychological sciences and social sciences https://pubmed.ncbi.nlm.nih.gov/15358792/	Neuropsychology	English-speakers; 236 initially aged 30–87 tested up to five times over 20 years (129 males)	Boston Naming Test	Regression modeling	Significant linear and quadratic change for lexical retrieval with age, although its decline is subtle (2% points per decade). People with initial high level of performance show less decline over time, supporting the cognitive reserve hypothesis.	DG-reserve LS-lifespan LS-reserve
Newman, R. S., & German, D. J. (2005). Life span effects of lexical factors on oral naming. Language and Speech https://pubmed.ncbi.nlm.nih.gov/16411502/	Behavioral	Teenagers, younger and older adults; 590 adolescents aged 12–19 (316 males), 358 adults aged 20–83 (160 males)	Picture naming (objects and actions), naming to open-ended sentences, naming to category exemplars	ANCOVA	Naming improves with maturation until adulthood and declines with aging. Older displayed similar effects of semantic and lexical and phonological properties as younger for naming, but the weighting of lexical factors appears to change with age. Age of acquisition and familiarity play a protective role for naming. Word frequency and form properties of words have similar effects in adulthood.	LS-lifespan
Brickman, A. M., Paul, R. H., Cohen, R. A., Williams, L. M., MacGregor, K. L., Jefferson, A. L., Tate, D. F., Gunstad, J., & Gordon, E. (2005). Category and letter verbal fluency across the adult lifespan: relationship to EEG theta power. Archives of clinical neuropsychology: the official journal of the National Academy of Neuropsychologists https://pubmed.ncbi.nlm.nih.gov/15939182/	Behavioral EEG	English/Dutch-speakers: 196 aged 21–30 (94 males), 87 aged 31–40 (53 males), 72 aged 41–50 (27 males), 61 aged 51–60 (27 males), 35 aged 61–70 (17 males), 20 aged 71–82 (13 males)	Alphabetic and categorical fluency task	ANOVA, chi-square, correlation	Phonological and semantic fluency decline linearly with age; greater rate of decline for semantic fluency. Theta power negatively correlates with age and positively with semantic fluency performance.	LS-lifespan
Zec, R. F., Markwell, S. J., Burkett, N. R., & Larsen, D. L. (2005). A longitudinal study of confrontation naming in the “normal” elderly. Journal of the International Neuropsychological Society : JINS. https://pubmed.ncbi.nlm.nih.gov/16248907/	Neuropsychology	English-speakers; 541 older aged 50–99 (189 males)	Boston Naming Test, Alzheimer Disease Assessment Scale	*t*-test, correlation, reliable change index, linear regression	Age-related changes for lexical retrieval are non-linear. Improvement in the 50s age, no change in the 60s age, and decline in the 70s and 80s age groups. An annual decline of at least 4 points on the BNT is necessary to conclude a reliable decline.	DG-reserve LS-reserve
Coppens, P., & Frisinger, D. (2005). Category-specific naming effect in non-brain-damaged individuals. Brain and Language https://pubmed.ncbi.nlm.nih.gov/15896384/	Behavioral Neuropsychology	English-speakers; 30 adults aged 20–30 (7 males), 30 adults aged 55–74 (9 males), 30 adults aged 77–92 (8 males)	MMSE, picture naming (living and non-living categories)	*t*-test, ANOVA	Errors increased in each group with age, mainly for living items. Older name better non-living than living items (not observed in younger). Progressive erosion of retrieval during aging affects differently representations of living and non-living concepts. Gender effect obtained for younger participants.	LS-lifespan
LaGrone, S., & Spieler, D. H. (2006). Lexical competition and phonological encoding in young and older speakers. Psychology and Aging https://pubmed.ncbi.nlm.nih.gov/17201499/	Behavioral Neuropsychology	English-speakers; 30 young adults and 30 older adults	Digit–Symbol subscale of Wechsler Adult Intelligence Scale (WAIS–III), WAIS Vocabulary subscale, picture naming task (objects)	Regression analysis, mixed-factor ANOVA	Name agreement and name frequency are significant predictors of picture naming performance in younger and older adults. Older are more influenced by the name agreement than younger. Competition for lexical selection may be an age-sensitive stage for lexical production.	LS-lifespan
Gollan, T. H., & Brown, A. S. (2006). From tip-of-the-tongue (TOT) data to theoretical implications in two steps: when more TOTs means better retrieval. Journal of experimental psychology - study 1 https://pubmed.ncbi.nlm.nih.gov/16846276/	Behavioral Neuropsychology	8 young adults (M = 20.89 y), 18 older adults (M = 77.28 y)	MMSE, WAIS–R Vocabulary subtest, TOT elicitation task (object naming)	ANOVA	Retrieval in older was accurate but slower. Older showed significantly more TOTs than younger but only for less familiar words. TOTs entail partially successful retrieval; hence, TOTs reflect better ability to access the lexicon, suggesting that age induces both age-positive and age-negative effects on lexical retrieval. Increased experience of older adults makes lexical representations more accessible, particularly for less familiar words.	DG-reserve LS-lifespan
Abrams, L., Trunk, D. L., & Merrill, L. A. (2007). Why a superman cannot help a tsunami: activation of grammatical class influences resolution of young and older adults' tip-of-the-tongue states. Psychology and aging, https://pubmed.ncbi.nlm.nih.gov/18179301/	Behavioral Neuropsychology	English-speakers; 60 adults aged 18–23; 60 adults aged 61–73 and 60 adults aged 75–89	MMSE, TOT elicitation task (general knowledge questions)	ANOVA	Age affects resolution of TOTs as a function of the prime’s grammatical class. Overall, phonology can influence lexical selection and mainly in oldest adults.	LS-reserve
Fogler, K. A., & James, L. E. (2007). Charlie Brown versus Snow White: The effects of descriptiveness on young and older adults' retrieval of proper names. The Journals of Gerontology Series B: Psychological Sciences and Social Sciences https://pubmed.ncbi.nlm.nih.gov/17673529/	Behavioral Neuropsychology	English-speakers; 20 aged 18–23, 20 aged 63–81	Shipley vocabulary test, MMSE, picture naming test (faces)	ANOVA, *t*-test	Older show more difficulties in retrieving names than young. Retrieval of non-descriptive names especially impaired in older adults. The age differences are smaller for descriptive names.	LS-lifespan
Shafto, M. A., Burke, D. M., Stamatakis, E. A., Tam, P. P., & Tyler, L. K. (2007). On the tip-of-the-tongue: neural correlates of increased word-finding failures in normal aging. Journal of cognitive neuroscience https://pubmed.ncbi.nlm.nih.gov/17892392/	Behavioral Neuropsychology MRI-VBM	English-speakers; 46 adults aged 19–88.	Raven’s Progressive Matrices (RPM), MMSE, TOT task, National Adult Reading Test, digit span forward and backward (Wechsler), standardized 40-item vocabulary test, Boston Naming Test, Edinburgh Handedness Inventory, TOT elicitation task (picture naming of famous faces)	GLM, multiple linear regression	TOTs frequency positively correlated with age and negatively correlated with gray matter atrophy of the left insula. Raven’s Progressive Matrices errors increased with age but the performance did not correlate with gray matter atrophy of the insula. Age-related atrophy of cerebral regions involved in phonological production would contribute to word production deficits with age.	LS-reserve
Strauss Hough M. (2007). Incidence of word finding deficits in normal aging. Folia phoniatrica et logopaedica: official organ of the International Association of Logopedics and Phoniatrics (IALP) https://pubmed.ncbi.nlm.nih.gov/17172782/	Neuropsychology	English-speakers; 50 aged 54–75 (25 males)	The Test of Adolescent/Adult Word Finding (TAWF), MMSE, Peabody Picture Vocabulary Test-Revised (PPVT-R), Western Aphasia Battery (WAB)	Hierarchical cluster analysis, k-means, correlation	One third of adults make errors for lexical retrieval. The most frequent error for nouns was production semantically related to the target. Three profiles were reported for naming reflecting the variability in terms of word retrieval in healthy aging.	LS-lifespan
Kavé, G., Samuel-Enoch, K., & Adiv, S. (2009). The association between age and the frequency of nouns selected for production. Psychology and aging https://pubmed.ncbi.nlm.nih.gov/19290734/	Behavioral Neuropsychology	Hebrew-speakers; 20 aged 20–29 (10 males), 23 aged 30–39 (11 males), 20 aged 40–49 (7 males), 21 aged 50–59 (11 males), 20 aged 60–69 (10 males), 32 aged 70–85 (11 males)	MMSE, picture naming (objects), semantic fluency task, picture description	Correlations, *t*-test	Aging decreases lexical production according to lexical frequency. Instead of selecting the most common nouns, older adults appear to produce less frequent nouns, most likely because of their larger vocabulary that might represent a compensatory mechanism with age.	DG-reserve LS-lifespan
Galdo-Alvarez, S., Lindín, M., & Díaz, F. (2009). The effect of age on event-related potentials (ERP) associated with face naming and with the tip-of-the-tongue (TOT) state. Biological psychology https://pubmed.ncbi.nlm.nih.gov/19428964/	Behavioral EEG	Spanish-speakers; 13 adults aged 19–24 (9 males), 10 adults aged 60–81 (7 males)	TOT elicitation task (picture naming of famous faces)	ERP, ANOVA	Older do not show ERP differences between correct and incorrect responses for picture naming compared to younger. Older show reduced ERP amplitude from the first item recognition stages onwards, reflecting difficulties in categorizing the stimulus. They showed wider and more frontal location of ERP components, associated with accessing identity, lexico-phonological processing, and revision of item categorization. These modifications would be compensatory mechanisms.	LS-lifespan LS-reserve
Galdo-Alvarez, S., Lindín, M., & Díaz, F. (2009). Age-related prefrontal over-recruitment in semantic memory retrieval: Evidence from successful face naming and the tip-of-the-tongue state. Biological psychology https://pubmed.ncbi.nlm.nih.gov/19559070/	Behavioral EEG	Spanish-speakers; 13 adults aged 19–24 (9 males), 10 adults aged 60–81 (7 males)	WAIS vocabulary, TOT elicitation task (picture naming of famous faces)	Temporal principal component analysis (tPCA), ERP, non-parametric statistical analysis (randomization tests)	Older adults do not show reduced brain activity compared to younger. They show additional prefrontal activation depending on the processing stage and experimental condition as well as on the success of retrieval. Overall, older activate the same regions as younger during successful retrieval, although to a greater extent, reflecting coping with increased retrieval demands.	DG-reserve
Shafto, M. A., Stamatakis, E. A., Tam, P. P., & Tyler, L. K. (2010). Word retrieval failures in old age: the relationship between structure and function. Journal of Cognitive Neuroscience https://pubmed.ncbi.nlm.nih.gov/19642890/	Behavioral Neuropsychology fMRI, VBM	15 adults aged 20–37, 14 adults aged 66–88.	MMSE, National Adult Reading Test, TOT elicitation task (picture naming of famous faces)	GLM, ROI-specific analysis, ANOVA	Age effects were observed during TOTs, younger but not older generating more activity of left insula, compared to successful naming. In older, lower levels of activity were observed during TOTs supporting the role of an age-related neural mechanism impacting older more than younger adults. Results support neural account of word retrieval with age although word production is not universally impacted by age. Atrophy reduces the ability to modulate neural responses and overcome the retrieval failures.	LS-lifespan LS-reserve DG-reserve
Hanna-Pladdy, B., & Choi, H. (2010). Age-related deficits in auditory confrontation naming. Psychology and aging https://pubmed.ncbi.nlm.nih.gov/20677880/	Behavioral Neuropsychology	English-speakers; 71 adults aged 30–35, 64 adults aged 60–85.	MMSE, American National Adult Reading Test, Boston Naming Test, Hearing Handicap Inventory for the Elderly, naming test under auditory, visual, and multisensory conditions	ANOVA, *t*-test	Older adults are less accurate and slower for naming. All participants were more impaired and slower while naming action sounds than pictures or audiovisual combinations. Older adults show lower accuracy and increased latencies for auditory naming. Multisensory enrichment facilitates lexical retrieval in older adults.	DG-reserve LS-lifespan
Obler, L. K., Rykhlevskaia, E., Schnyer, D., Clark-Cotton, M. R., Spiro, A., 3rd, Hyun, J., Kim, D. S., Goral, M., & Albert, M. L. (2010). Bilateral brain regions associated with naming in older adults. Brain and language https://pubmed.ncbi.nlm.nih.gov/20399492/	Neuropsychology VBM, DTI	MRI, 21 adults aged 56–79 (12 males); DTI, 21 adults aged 56–79 (12 males)	Boston Naming Test and the Action Naming Test	Linear regression, tract-based spatial statistics, GLM	Older adults with better naming performance rely on right-hemispheric peri-sylvian regions in conjunction with left-hemispheric, as well as on mid-frontal regions. Older with better performance for lexical retrieval also activate prefrontal regions related to executive functions, more anteriorly than frontal regions involved in language and right-hemisphere peri-Sylvian and mid-frontal regions. VBM of white matter indicates increased density in older adults with greater performance for naming.	LS-lifespan DG-reserve LS-reserve
Kavé, G., Knafo, A., & Gilboa, A. (2010). The rise and fall of word retrieval across the lifespan. Psychology and aging https://pubmed.ncbi.nlm.nih.gov/20853975/	Behavioral Neuropsychology	Hebrew-speakers; 50 child participants aged 5–17, 695 adults aged 18–86, (276 participants above 60 years old)	MMSE, Hebrew naming test	Regression, correlation, ANOVA, ANCOVA	Early-life rise for naming is followed by a late-life fall. A significant change in naming abilities occurs only late in life, into the eighth’s decade. The increase in naming during childhood is steeper than its decrease in late adulthood. The large and rich lexicon available in older adults helps to overcome temporary deficiencies in word access. Naming difficulties related to age appear to be induced by difficulty to access the existing knowledge.	LS-lifespan
Farrell, M. T., & Abrams, L. (2011). Tip-of-the-tongue states reveal age differences in the syllable frequency effect. Journal of experimental psychology. Learning, Memory, and Cognition https://pubmed.ncbi.nlm.nih.gov/21244118/	Behavioral Neuropsychology	English-speakers; 79 adults aged 18–26, 110 adults aged 60–74, and 86 adults aged 75–89	MMSE, vocabulary, forward and backward digit span, TOT elicitation task (definition-based general knowledge questions) with priming condition	ANOVA, *t*-test	Old and very old adults experience more TOTs for words beginning with low-frequency first syllables than for words beginning with high-frequency first syllables. Syllable frequency effect is explained by phonological and lexical mechanisms and shows the importance of considering sub-lexical frequency when evaluating lexical production in aging.	LS-lifespan LS-reserve
Stamatakis, E. A., Shafto, M. A., Williams, G., Tam, P., & Tyler, L. K. (2011). White matter changes and word finding failures with increasing age PloSone https://pubmed.ncbi.nlm.nih.gov/21249127/	Behavioral Neuropsychology DTI	English-speakers; 28 adults aged 19–82 (M = 52.4)	Edinburgh Handedness Inventory, MMSE, screening test for dementia, National Adult Reading Test, digit span forward and backward, Shipley vocabulary test, Boston Naming Test, TOT elicitation task (picture naming of famous faces)	Correlation, multiple linear regression	Widespread changes in fractional anisotropy (FA) with age and positive correlation of FA with successful retrieval throughout the white matter structures supporting cortical gray matter language network. TOT increase with age is related to deterioration of the integrity of superior longitudinal fasciculus (SLF). There were left-right hemispheric asymmetries in terms of FA, which remained stable throughout the lifespan. Despite a global FA reduction, there would be sufficient residual WM integrity to support age-related bilateral modifications. An anteroposterior gradient of FA was observed with decrease for fronto-temporal and sparing of occipital regions.	LS-lifespan
Facal, D., Juncos-Rabadán, O., Rodríguez, M. S., & Pereiro, A. X. (2012). Tip-of-the-tongue in aging: influence of vocabulary, working memory and processing speed. Aging clinical and experimental research https://pubmed.ncbi.nlm.nih.gov/22960259/	Behavioral Neuropsychology	Spanish-speakers; 36 adults aged 19–26 (19 males); 33 aged 50–59 (16 males); 33 aged 60–69 (13 males); 31 aged 70–82 (15 males)	MMSE, TOT task, Vocabulary subscale of the Wechsler Adult Intelligence Scale, Peabody Picture Vocabulary Test, Reading Span task, Operation Span with words, Counting Span, Test of Attentional Performance, tonic alertness, phasic alertness, and visual attention tasks	ANOVA and post hoc Scheffe tests, bivariate correlations, exploratory factorial analysis (EFA), confirmatory factorial analysis (CFA), structural equation modeling (SEM)	Aging associated with high frequency of TOTs, mainly for proper names, better performance for vocabulary, poorer working memory performance, and reduction of processing speed. Increased TOT frequency is negatively modulated by slowed processing. Increased performance for vocabulary tests is positively modulated by working memory. No relation between vocabulary and TOTs.	DG-reserve
Gordon, J. K., & Cheimariou, S. (2013). Semantic interference in a randomized naming task: effects of age, order, and category. Cognitive neuropsychology https://pubmed.ncbi.nlm.nih.gov/24499271/	Behavioral Neuropsychology	English-speakers; adults aged 22–89 (60 males)	WAIS-IV vocabulary scaled score, the Boston Naming Test, Object & Action Naming Battery, Philadelphia Naming Test, Snodgrass-and-Vanderwart-like pictures	Mixed-effects linear regression	Age had a slowing effect on reaction times for picture naming. Older adults are not more susceptible to semantic interference than younger.	DG-reserve
Verhaegen, C., & Poncelet, M. (2013). Changes in naming and semantic abilities with aging from 50 to 90 years. Journal of the International Neuropsychological Society: JINS https://pubmed.ncbi.nlm.nih.gov/23237304/	Behavioral Neuropsychology	French-speakers; 30 adults aged 25–35 (14 males), 30 adults aged 50–59 (8 males), 30 adults aged 60–69 (14 males), 30 adults above 70 (14 males)	Mattis Dementia Rating Scale, Mill–Hill test, picture naming task (objects), odd/even judgment task, Pyramids and Palm Trees test, synonym judgment task	ANOVA, correlation	Participants in their 50s show a decline in naming performance with increased latencies. Participants in their 60s and 70s showed both decrease in accuracy and increased latency. Only participants above 70 years of age showed semantic impairment. Results suggest age-related decline at a semantic level of the language system.	DG-reserve LS-lifespan
Zhang, H., Sachdev, P. S., Wen, W., Kochan, N. A., Crawford, J. D., Brodaty, H., Slavin, M. J., Reppermund, S., Kang, K., & Trollor, J. N. (2013). Grey matter correlates of three language tests in non-demented older adults. PloS one https://pubmed.ncbi.nlm.nih.gov/24224044/	Behavioral, VBM	English-speakers; 205 adults aged 70–79 (97 males), 139 very old adults aged 80–90 (60 males)	Controlled Oral Word Association Task (COWAT), Category Fluency (CF), Boston Naming Test	Correlation, conjunction analysis	Word generation to phonemic cues (COWAT) correlated with right frontal and left temporal gray matter (GM) volume. Semantic fluency correlated with left frontal and left temporal GM volume. Picture naming correlated with bilateral temporal GM volume. Reduced hemispheric asymmetry in terms of GM for regions related to these tests. Older participants show stronger correlation between structural laterality indices and language performance compared to very old participants, suggesting variable patterns of language lateralization in stages of late life.	DG-reserve LS-lifespan LS-reserve
Manenti, R., Brambilla, M., Petesi, M., Miniussi, C., & Cotelli, M. (2013). Compensatory networks to counteract the effects of ageing on language. Behavioural brain research https://pubmed.ncbi.nlm.nih.gov/23602922/	Behavioral Neuropsychology TMS	Italian-speakers; 13 adults aged 65–78 (4 males)	MMSE, Raven’s Coloured Progressive Matrices, Token Test, verbal fluency (phonemic and semantic), Story Recall, Rey Osterrieth Complex Figure Recall, Digit Span, Spatial Span, Copy, Trail Making Test A and B, Battery for Analysis of Aphasic Deficits, picture naming task (objects and actions)	*t*-test, correlation	Differential left-right involvement of dorsolateral prefrontal cortex (DLPFC) during action naming was in association with higher accuracy and faster responses. For low-performer older adults: DLPFC left > right suggesting similar network as younger adults, but recruited inefficiently. For high-performer older adults: no DLPC asymmetry in association with better phonemic fluency. Successful aging would be associated with less prefrontal asymmetry as an efficient strategy for counteracting age-related decline of lexical retrieval.	DG-reserve
Marsolais, Y., Perlbarg, V., Benali, H., & Joanette, Y. (2014). Age-related changes in functional network connectivity associated with high levels of verbal fluency performance. Cortex https://pubmed.ncbi.nlm.nih.gov/25014614/	Behavioral fMRI	French-speakers; 14 adults aged 20–31 (7 males) and 14 adults aged 60–73 (6 males)	MMSE, Edinburgh Handedness Inventory, Stroop Victoria Test (SVT), Trail Making Test (TMT, parts A and B), Alpha-span task, verbal fluency (four semantic and four phonological)	ICA, exploratory network detection procedure (NEDICA), hierarchical measures of functional network integration, *t*-test	Aging affects functional integration of cortical networks without disrupting lexical speech production abilities in high-performing older adults. A task demand/age interaction was found for functional connectivity within the anterior and posterior subnetworks of verbal fluency network. Local changes in functional integration among areas supporting lexical production are modulated by age and task demands.	LS-lifespan LS-reserve
Marsolais, Y., Methqal, I., & Joanette, Y. (2015). Marginal neurofunctional changes in high-performing older adults in a verbal fluency task. Brain and Language https://pubmed.ncbi.nlm.nih.gov/25461916/	Behavioral fMRI	French-speakers; 14 adults aged 20–31 (7 males); 14 adults aged 60–73 (6 males)	MMSE, Edinburgh Handedness Inventory, Stroop Victoria test (SVT), Trail making test (TMT, parts A and B), Alpha-Span task; verbal fluency tasks (four semantic and four phonological)	GLM, *t*-test, conjunction analysis, between-group ROIs analyses	Semantic and phonological fluency reduction in older is only marginally accompanied with neurofunctional changes. Speech production abilities involved in semantic and phonological verbal fluency remain relatively stable in healthy older adults who have high level of education. Negative correlation between processing speed and semantic fluency performance in older adults.	DG-reserve LS-lifespan LS-reserve
Lee, S. H., Kim, H., Kim, J., Yoon, J. H., & Kim, S. R. (2015). Initial phase performance in a 30-s verbal fluency task as being reflective of aging effect. Geriatrics & gerontology international https://pubmed.ncbi.nlm.nih.gov/24730516/	Neuropsychology	Korean-speakers; 119 aged 60–84 (27 males)	MMSE, semantic fluency (animal category)	ANOVA, MANOVA	The number of words produced gradually declines with age. Significant difference in performance among age groups in three phases (0–5, 6–10, 16–20 s) of semantic fluency task, with older groups showing the worst performance. The first word production within the first 5-s phase is significantly delayed in oldest adults. The difficulty in semantic-lexical retrieval and progressive slowing down of task processing reflect deterioration of attention and processing speed during aging.	DG-reserve LS-lifespan LS-reserve
Baciu, M., Boudiaf, N., Cousin, M., Perrone-Bertolotti, M., Pichat, C., Fournet, N., Chainay, H., L Lamalle, L., Krainik, A. (2016). Functional MRI evidence for the decline of word retrieval and generation during normal aging. Age (Dordrecht, Netherlands) https://pubmed.ncbi.nlm.nih.gov/26711670/	Behavioral Neuropsychology fMRI	French-speakers; 16 adults aged 30–59 (11 males), 14 adults aged 60–84 (10 males)	Picture naming, picture categorization (Pyramid Palm and Tree Test), semantic fluency; vocabulary and verbal intelligence, Mill–Hill vocabulary scale; verbal automatisms; Trail Making Test A-B; Digit Span Memory; Frontal Assessment Battery, MMSE; HAD; Dubois’ episodic memory test; McNair questionnaire; Poitrenaud Questionnaire, Edinburgh Handedness	GLM, lateralization indices, *t*-test, correlation analysis	Older have difficulties to access lexico-semantic representations by a slowdown of executive functions, without any conceptual loss. In addition, there was lower speed for executive functions, tendency to reduced verbal fluency, and frequent automatisms; fMRI showed atypical patterns of brain networks for lexical production, compared to younger adults.	DG-reserve LS-lifespan
Hoyau, E., Boudiaf, N., Cousin, E., Pichat, C., Fournet, N., Krainik, A., Jaillard, A., & Baciu, M. (2017). Aging Modulates the Hemispheric Specialization during Word Production. Frontiers in aging neuroscience https://pubmed.ncbi.nlm.nih.gov/28536520/	Behavioral fMRI	French-speakers adults; 13 younger adults (M = 40.07, SD = 8.33, 8 males) 14 older adults (M = 71.21, SD = 6.93, 11 males)	MMSE, Poitrenaud questionnaire, Edinburgh Handedness Inventory, Hospital Anxiety and Depression test (HAD), episodic memory deficits (5-words test), semantic verbal fluency, the verbal automatisms test, Mill–Hill B vocabulary scale, forward and backward digit span tests, MacNair questionnaire, Trail Making Test (TMT-A and TMT-B), picture naming task DO-80 (objects)	Mann–Whitney tests, GLM, *t*-test, ROI analysis, ANCOVA	Aging effect consisting of supplementary activation of left posterior (temporo-parietal) regions in older and asymmetric activation along the left fronto-temporal axis, reflecting enhanced recruitment of semantic knowledge in older adults to maintain correct accuracy for naming. Bilateral recruitment of frontal regions to maintain appropriate response times especially in older adults who are faster performers, reflecting compensatory executive-based mechanisms in relation to the cognitive reserve.	DG-reserve LS-lifespan
Shafto, M. A., James, L. E., Abrams, L., Tyler, L. K., & Cam-CAN (2017). Age-Related Increases in Verbal Knowledge Are Not Associated With Word Finding Problems in the Cam-CAN Cohort: What You Know Won't Hurt You. The journal of gerontology, Series B, Psychological Sciences and Social sciences https://pubmed.ncbi.nlm.nih.gov/27371482/	Behavioral Neuropsychology	178 adults aged 18–39, 280 adults aged 40–64, 250 adults aged 65–88	Spot-the Word Test (STW), Cattell Culture Fair, Scale 2 Form A, TOT elicitation task (picture naming famous faces), a picture naming task (objects)	Correlation, linear regressions	Age and crystallized intelligence predict independently the TOTs, with higher TOTs for older and for participants with lower crystallized intelligence scores. Similar relationships were found for picture naming accuracy. Decline of word retrieval is not associated with lifelong increases of verbal knowledge. Potentially compensatory role of increased lexical knowledge for word retrieval in older adults.	DG-reserve LS-lifespan
Lorenz, A., Regel, S., Zwitserlood, P., & Rahman, R. A. (2018). Age-related effects in compound production: Intact lexical representations but more effortful encoding. Acta Psychologica https://pubmed.ncbi.nlm.nih.gov/30404741/	Behavioral Neuropsychology	German-speakers; 30 young (9 males; M= 27, SD = 4.9); 30 old (9 males; M = 70.5, SD = 4.3)	MMSE, Multiple Choice Vocabulary Intelligence test, version B, picture naming task (objects) with interference (morphologically and semantically); non-verbal attention control Simon task	GLM	Slower picture naming and less accurate responses in older adults. Older speakers showed stronger morphological facilitation. Semantic distractor effects unaffected by age. Non-verbal attentional control declines with age. Picture naming difficulties independent from the attentional control and reduced morpho-phonological encoding in older.	DG-reserve LS-lifespan
Boudiaf, N., Laboissière, R., Cousin, É., Fournet, N., Krainik, A., & Baciu, M. (2018). Behavioral evidence for a differential modulation of semantic processing and lexical production by aging: a full linear mixed-effects modeling approach. Neuropsychology, Development, and Cognition https://pubmed.ncbi.nlm.nih.gov/27883290/	Behavioral Neuropsychology	French-speakers; 72 adults aged between 30–84 (35 males)	Picture naming, picture categorization (Pyramid Palm and Tree Test), numerical judgment (parity judgment task), color judgment (CJ); vocabulary and verbal intelligence, Mill–Hill vocabulary scale; verbal automatisms; Trail Making Test A-B; Digit Span Memory Test; Frontal Assessment Battery, Mini Mental State Evaluation (MMSE), Hospital Anxiety and Depression scale (HAD), Dubois’ episodic memory test; McNair questionnaire of memory and language complaints in daily life, Poitrenaud Questionnaire, Edinburgh Handedness Inventory	GLM	Naming is more automatic and semantic processing becomes more difficult with age, with nonspecific general slowdown of cognitive processing. Accuracy of lexical production remains unaltered with age, based on compensatory automatic processes. A slowdown of semantic processing may occur in normal aging.	DG-reserve LS-lifespan
Hoyau, E., Roux-Sibilon, A., Boudiaf, N., Pichat, C., Cousin, E., Krainik, A., Jaillard, A., Peyrin, C., & Baciu, M. (2018). Aging modulates fronto-temporal cortical interactions during lexical production. A dynamic causal modeling study. Brain and Language https://pubmed.ncbi.nlm.nih.gov/29913316/	Behavioral fMRI	French-speakers; 15 adults aged 30–56 (10 males), 14 adults aged 59–85 (11 males)	MMSE, Edinburgh Handedness Inventory, Hospital Anxiety and Depression test (HAD), episodic memory deficits (5-words test) picture naming task DO-80 (objects)	GLM, dynamic causal modeling (DCM), *t*-test	In older adults, there is a bi-directional interaction between inferior frontal and medial temporal cortices, but not between inferior frontal and lateral temporal cortices. Older adults use new strategy with supplemental access to concepts and semantic retrieval processes. Compensatory mechanisms for lexical production in older adults are based on increased semantic memory access under the influence of top-down inferior frontal to medial temporal cortices (including the hippocampus).	LS-lifespan
Mohan, R., & Weber, C. (2018). Neural activity reveals effects of aging on inhibitory processes during word retrieval Neuropsychology, development, and cognition. Section B, Aging, Neuropsychology and Cognition https://pubmed.ncbi.nlm.nih.gov/30223706/	Behavioral EEG	English-speakers; 15 older adults (10 females; 60.1–74.8 years), 13 middle-aged adults (13 females; 41.7–55 years), 15 young adults (11 females; 20–30.3 years)	Hollingshead education scale, Cognitive–Linguistic Quick Test, Digit Span Backward (DSB) subtest of the Wechsler Memory Scale-III, the Stroop Color–Word Test, Peabody Picture Vocabulary Test, Fourth Edition (PPVT-4), Test of Adolescent/Adult Word Finding (TAWF), picture naming (objects), ERP primed naming task (pseudo-word primes)	ANOVA	Normal activation of phonological processing during picture naming but age-related delays in inhibition of primed competitors. High correlation between N2 peak latency and clinical measures of inhibition suggested an age-related delay in the inhibition of primed lexical competitors which can begin at middle age.	DG-reserve LS-lifespan
Lorenz, A., Zwitserlood, P., Regel, S., & Abdel Rahman, R. (2019). Age-related effects in compound production: Evidence from a double-object picture naming task. Quarterly Journal of Experimental Psychology https://pubmed.ncbi.nlm.nih.gov/30269664/	Behavioral Neuropsychology	German-speakers; 32 young participants aged 18–34 (11 males), 32 older participants aged 65–77 (10 males)	MMSE, spot-a-word task, picture naming interference task with compound targets, visual-spatial version of the Simon task	Linear mixed models (LMMs), logit mixed-effects models	Older participants are slower and produced more errors than younger participants. Morphological effects of first-constituent distractors were stronger for older, semantic effects were not affected by age. Non-verbal attentional control processes contributed to morpho-phonological priming in older adults but no effect of semantic interference or semantic facilitation was observed. Word-finding difficulties in older result from deficient phonological encoding, whereas lexical-semantic and morpho-phonological representations seems to remain stable with age.	LS-reserve
Higby, E., Cahana-Amitay, D., Vogel-Eyny, A., Spiro, A., 3rd, Albert, M. L., & Obler, L. K. (2019). The Role of Executive Functions in Object- and Action-Naming among Older Adults. Experimental Aging Research https://pubmed.ncbi.nlm.nih.gov/31216948/	Neuropsychology	English-speakers; 305 middle-aged and older adults aged 55–84 (154 males)	MMSE, the Boston Naming Test (BNT), Action Naming Test (ANT), Trail Making Test, Alternating Category fluency task, phonemic and semantic verbal fluency, modified Stroop task, Stop Signal Paradigm, listening span, digit ordering, and month ordering, the Choice Reaction Time (CRT) task, Letter and Pattern Comparison tasks	Correlations, multiple regressions	Executive functions predict naming speed and accuracy in older adults. Shifting process predicts naming accuracy for object and action naming. BNT response times are lower with advancing age for adults with poorer shifting ability but remain stable for those with better shifting ability. Fluency predicted naming accuracy. Inhibition process did not contribute to naming accuracy or latencies in naming. Cognitive mechanisms underlying executive functions and those recruited for successful naming are related in older adults. Declines in naming performance with age may be partly due to declines of executive functions.	DG-reserve
Gertel, V. H., Karimi, H., Dennis, N. A., Neely, K. A., & Diaz, M. T. (2020). Lexical frequency affects functional activation and accuracy in picture naming among older and younger adults. Psychology and Aging https://pubmed.ncbi.nlm.nih.gov/32191059/	Behavioral fMRI	English-speakers; 30 younger adults aged 18–34 (14 males); 30 older adults aged 60–79 (13 males)	MMSE, Geriatric Depression Scale, Stroop Task, phonemic and categorical verbal fluency, vocabulary subtest of the Wechsler Adult Intelligence Scale (WAIS-III), reaction time (RT) task (shape detection) and a choice RT task, the California Verbal Learning Test (immediate and delayed recall, word recognition), adapted non-verbal working memory task, picture naming task (objects)	Logistic mixed-effect regression analysis, FMRIB’s local analysis of mixed effects, ANOVA	Younger and older respond less accurately for low-frequency words. Older show less accuracy than younger. Less frequent items require more cognitive control and perceptual resources, independently from age. Older and younger elicit a similar level of activation for high-frequency words. Compared to younger, older engage less key language (insula) or cognitive control (superior frontal, cingulate) regions when demand increases (low-frequency words). Younger rely on core language regions, older recruit both language network and regions outside this network, suggesting reduce segregation with age. Older adults who recruit less extra-language regions are able to name pictures more accurately.	Other than LARA-based (dedifferentiation)
Ouyang, M., Cai, X., & Zhang, Q. (2020). Aging Effects on Phonological and Semantic Priming in the Tip-of-the-Tongue: Evidence From a Two-Step Approach. Frontiers in Psychology https://pubmed.ncbi.nlm.nih.gov/32174876/	Behavioral Neuropsychology	Mandarin-speakers; 40 young adults aged 18–28, 42 old adults aged 60–72 years	Montreal Cognitive Assessment Scale (MoCA), picture naming task (faces), picture naming task (faces) with primes (2 types of semantic relatedness and 3 types of phonological relatedness), Stroop	ANCOVA	Older report TOTs for naming more frequently when retrieval is based on phonology. Young and old show comparable naming performance when based on semantics. For semantic-based deficit retrieval: larger interference for bottom-up semantic priming and smaller bottom-up phonological priming in older. For phonological-based retrieval deficit: age-related difference in bottom-up phonological but not in bottom-up semantic priming. Semantic representations would be richer with age but their connections with phonological representations are reduced.	LS-lifespan LS-reserve

Thereafter, we remind the main mechanisms underlying lexical production decline with aging.

## Mechanisms involved in lexical production decline with aging

Regarding language-specific mechanisms, the difficulty to produce a word can first be explained by difficulty to access conceptual and semantic representations [22, 84, 198], even if some authors argue for an additional disruption in semantic processing [46, 195]. Finding a word can also be imperiled by difficulties to retrieve or activate its phonological representation [2, 45]. Based on the node structure theory (NST; [114]), the transmission deficit hypothesis [116, 187] provides a model of age-related activation impairment. According to the NST, linguistic information is stored in interconnected nodes within a large hierarchically organized network with two crucial levels, a phonological system representing the sounds and metrical structure of words and a semantic system representing conceptual and lexical information about words. Language production is assumed to be influenced by the amount and speed of priming transmission across connections between nodes. Priming is a form of excitation that prepares a node for activation, allowing retrieval of the information represented by the node. Under the transmission deficit hypothesis, the strength of connections between nodes would become weaker with age, which would produce priming transmission deficits. Transmission deficits from semantic to phonology can impair activation of phonological nodes, leading to word retrieval failure [1, 25, 90, 203]. This hypothesis is supported by neuroimaging data collected by Shafto et al. [168]. They showed that the reduced ability of older adults to find words is related to neural atrophy in regions critical for phonological processes, which decreases the flexibility of the word production network. Overall, these findings suggest that two complementary language-specific mechanisms, one lexico-semantic and another one phonological, can explain age-related difficulties to produce words.

As mentioned above, other non-linguistic or domain-general mechanisms can explain lexical production difficulties in older adults, typically a decline in executive functions [76, 81, 204], together with reduced processing speed or not [156]. The processing speed theory attributes cognitive decline to a decrease in information processing speed, which results in operations prevented from being successfully executed or chained due to limited time [155]. The links between processing speed decrease and cognitive inhibition deficits have been debated [75, 113, 158]. Some studies suggest that naming deficits can be explained by difficulties to select an appropriate word among a set of similar alternatives [9, 101] or to inhibit non-pertinent competitors [67, 131]. A decline in attention and working memory has also been incriminated [27, 104, 130] as a potential source of word production deficit. Altogether, the studies reviewed above reveal that uncovering the mechanisms responsible for cognitive aging is challenged by the facts that age has heterogeneous effects on different cognitive processes and that cognitive aging does not manifest uniformly in the general population.

## Universal vs. idiosyncratic aging

Even though a universal decline in specific cognitive functions is manifest in all individuals as they grow older, the degree of decline varies from one individual to another. Comparison between older and younger groups is the commonly used approach to assess the effect of age on cognitive processes. It is generally observed at a group level that behavioral and cognitive performance scores in older adults are lower when compared to younger groups, reflecting a *universal* effect of age. However, among the older adults, a significant inter-individual variability may be observed, reflecting a non-uniform or *idiosyncratic* effect of age, some aging adults being cognitively more efficient than others and for longer periods [28, 32, 185, 191]. Understanding this variability is particularly important for identifying risk and preventive factors associated with cognitive aging, in order to promote successful aging. More specifically, healthy aging can in fact be described as either successful or usual/common [152, 153]. Successful aging, also called optimal health or super-aging [184], is associated with a low probability of disease or disability, high cognitive and physical function, and active engagement in social activities, while usual/common aging describes good cognitive and physical performance levels but higher risks of disease or disability [152]. Even if the theoretical concept of successful aging has been questioned because of the difficulty to find a consensual definition and implementation [36, 120], it offers a more positive prospect on aging and has opened a line of research focusing on the biological and lifestyle factors that may favor a more performant aging [10, 200].

### Cognitive aging, reserve, and compensatory mechanisms

The inter-individual variability in aging is generally explained by the notion of reserve [182] with two complementary dimensions [13, 183], one passive or cerebral [92, 159] and another active or cognitive [182]. The cerebral reserve is described as the amount of brain deterioration that can be tolerated before reaching a critical threshold, above which functional consequences are unavoidable. On the other hand, cognitive reserve, an active mechanism which may explain some inter-individual variability, refers to the ability to efficiently use the available cerebral reserve to perform a specific task. Two implementations of the cognitive reserve have been proposed, neural reserve, i.e., the optimized use of typical predefined networks for a given cognitive process, and neural compensation, i.e., the use of alternative networks than those classically predefined for a specific cognitive process [13, 183]. Large cognitive reserve suggests more flexible cerebral networks and greater ability to adjust behavior to the task constraints [15].

Cognitive reserve, be it neural reserve or neural compensation, is related to the reorganization or plasticity of cerebral networks, either intrinsic, which are observed in the absence of cognitive tasks (rest), or extrinsic, which are required by cognitive tasks. In the absence of any specific task, the intrinsic activity is represented by several distinct and distributed functional subnetworks or modules, involving several brain regions which are connected with each other [205]. Among these modules, one can mention the default mode network (DMN; [123, 143, 172]), executive control, salience, sensorimotor, and visual networks [109, 176]. The modular organization may offer a protective mechanism in the case of injury [58]. Moreover, these intrinsic networks evolve under the effect of age toward a decrease in network segregation, as has been observed in older participants [29, 63]. A recent longitudinal study [117] performed on a 4-year period showed significant reconfiguration of these networks in healthy older adults, with significant network flexibility between modules, even if the authors did not find a correlation with cognitive performance. Other studies focused on variations in the activity of the default mode network with age. The DMN is associated with a variety of functions such as semantic processing [19, 206], mind-wandering [121] and, more generally, with internally generated cognitive operations [4, 72]. With normal aging, DMN activity is modified in terms of either reallocation of activity within DMN regions [125, 146] or changes in the magnitude of DMN deactivation [21]. Another line of research has revealed disrupted functional connectivity (FC) within the DMN and reorganizations of the connectivity between and within large-scale cognitive networks [154]. Specifically, FC decreases with age [5, 38, 50, 69, 202, 209] and older individuals show reduced brain connectivity [5, 70]. In the same line, Li et al. [108] showed modifications of functional and structural connectivity reflecting increased bilateral prefrontal recruitment, as a compensatory mechanism that may counteract age-related unilateral efficiency decline. Krieger-Redwood et al. [100] showed that behavioral changes with age for a semantic control task are correlated with measures of intrinsic connectivity between the anterior temporal lobe and medial prefrontal cortex within the DMN. Authors showed that compared to younger individuals, older adults showed reduced connectivity between the right anterior temporal lobe and medial prefrontal cortex and this decrease in connectivity was correlated with preserved verbal semantic performance but reduced semantic retrieval control. The patterns of functional changes in prefrontal regions implicated in cognitive control together with modifications in the DMN led Spreng and colleagues to propose the DECHA model (Default-Executive Coupling Hypothesis of Aging; [180, 181, 194]). In the DECHA model, it is claimed that older adults fail to modulate functional connectivity between executive control regions and the DMN in response to increasing task challenge, relying more on stored semantic knowledge (DMN) and less on executive control. This would be reflected in reduced DMN suppression during goal-directed tasks and an increased inflexible coupling with lateral prefrontal executive networks, in older adulthood. In the same line, Muller et al. [127] found that a finely coordinated interaction between DMN, executive-control, and language networks is crucial for successful verbal fluency performance in older adults. Furthermore, He et al. [77] quantified dynamic reconfiguration of several resting state functional networks in adults of different ages and showed differential modifications of segregation and integration with age. Reduced dynamic segregation was observed for all networks with age, except for the cerebellum. Aging increased dynamic integration of several intrinsic networks, such as the DMN, FPN (fronto-parietal network), and visual network. According to the authors, these findings suggest a significant modulation of the intrinsic activity of aging brain networks, reflecting age-related adaptive dedifferentiation (reduced neural specificity) and compensation mechanisms. This might contribute to maintaining cognitive performance through the modulation of cognitive reserve. These findings are in line with other studies which found increased between-network connectivity and reduced within-network connectivity [29, 31, 95], supporting the idea of increased integration with compensation and reduced segregation with dedifferentiation during aging.

Other brain reorganization patterns have been observed in older adults, at an intra-hemispheric level, such as the Posterior-Anterior Shift in Aging (PASA) model [39, 42] assuming reduction of activity of posterior occipito-temporal regions with increased frontal activity. Hoyau, Roux-Sibilon, et al. [84] examined if the compensatory strategies associated with the effect of age on the effective connectivity of lexical production networks would pertain to a neural reserve mechanism (reflected by increased connectivity between the medial temporal cortices including the hippocampus and lateral temporal cortices), or to a neural compensation mechanism (reflected by increased connectivity between the inferior frontal cortex and medial temporal cortex). While younger adults showed bi-directional interaction between left inferior frontal and left temporal cortices, suggesting recruitment of lexico-semantic representations, older adults showed bi-directional interaction between inferior frontal and medial temporal cortices, but not between inferior frontal and lateral temporal cortices. These results suggested that older adults develop compensation strategies facilitated by top-down mechanisms from inferior frontal to medial temporal cortices. Another compensatory mechanism observed in older adults is described by the HAROLD (Hemispheric asymmetry reduction in older adults) model of cognitive aging. Namely, during the performance of various cognitive tasks, older participants show a reduction of hemispheric asymmetry in comparison to younger adults [28]. This pattern is especially patent in high-performing older adults [118]. It could represent the use of either cerebral or cognitive reserve, at regional or network levels.

### Aging-modulatory reserve factors (AMF)

Available evidence on inter-individual variation in vulnerability to cognitive decline [91] reveals resilience mechanisms that need to be better understood in order to prevent pathological aging and neurodegenerative conditions.

In recent studies, more attention is drawn to aging-modulatory reserve factors (AMF), various elements that can modulate reserve, either increasing it or acting as a potential risk for pathological aging. In general, aging can be described as a result of the interaction between genetic and environmental factors [96]. In the case of damage or tissue loss and reduced functionality of organs, this interaction will manifest through a decrease in performance, including cognitive decline.

The notion of consilience, the independent converging agreement of explanations at various levels, supports the theory construction of health and by extension, to cognition. In the domain of aging, it might suggest that the effects of a specific modulatory factor are exerted at various levels, from molecular genetics, epigenetics, sub-systems to higher complex and dynamic interactions between cognitive systems. Indeed, a specific modulatory factor may affect the relations between cerebral functions from perception to high-level cognitive processes, from a molecular to a cerebral and cognitive level, in tight relation with social and environmental factors (for details, see [163]).

It is beyond the scope of the present paper to provide an exhaustive description of studies on modulatory factors of cognitive reserve and normal aging. Therefore, in Supplementary Table (Annex), we present a synthesis of main studies on AMF and their relation with cognitive aging. This synthetic presentation of main studies was conducted using the PubMed database during November 2020 for a systematic literature review of age-related lexical changes in lexical production. Keywords used were “aging/ageing”, “reserve”, “healthy”, “cognitive” in the following combination: (aging OR ageing) AND reserve AND healthy AND cognitive. Two of the authors first screened the titles and abstracts of the resulting papers to assess their eligibility and then performed full-text scans to determine whether papers met the inclusion criteria. To be included in the review, the studies had to fulfill the following inclusion criteria: published between 2000 and 2020, written in English, the study includes older participants (60+), and the study specifically assesses the modulatory factors of the cognitive reserve during aging (i.e., not just effects of the cognitive reserve). The studies were left out if they fulfilled one of the following exclusion criteria: participants suffered from some form of pathology, results reported on very low statistical level (i.e., *p* < 0.1), and the majority of participants were middle-aged (<60). Case studies, meta-analysis, and review papers were excluded. Through this process, 48 papers were identified, some of them (in green) being mentioned several times, for several tested AMF. In the next section, we describe the main mechanisms considered to be involved in lexical production decline.

*Genetic predisposition and biological factors* play a significant role in the neuroplasticity observed during aging. For instance, the catechol-O-methyltransferase (COMT) genetic polymorphism has been brought up. Indeed, carrying less favorable combinations of alleles leads to poorer cognitive performance during aging [48, 122, 192]. Similarly, carrying the ε4 variant of ApoE is a risk factor for Alzheimer’s disease and this variant seems to be more frequently associated with increased risks for poorer functional status ([3]; see Supplementary table for specific relationsof the APOE-ε4 with cognitive activities) and reduced cognitive performance ([24, 44] cited by [175]). Positive associations have been found between social activities and the ADRB2 genotypes, with a favorable effect on health and longevity [208]. Moreover, the brain-derived neurotrophic factor (BDNF; see Supplementary Table) gene and expression of BDNF protein are particularly important for cognitive aging. The BDNF has a significant effect on the brain and cognition, based on synaptic plasticity, neurogenesis, memory, and neuronal stress resistance (see [119] cited by [163]). It has been observed greater cognitive decline in older individuals carrying less favorable combinations (Met variants) of alleles [49, 94, 136]. We mentioned above the stress as a significant factor that may affect global health and cognition. The stress would target the level of BDNF. BDNF is a potent neurotrophic factor and its reduced levels were identified in normal aging and neuropathological conditions (e.g. Alzheimer’s disease) in which its downregulation was related with neuronal atrophy and death [129]. Chronic stress can contribute to age-related changes by decreasing hippocampal BDNF expression [174]. Other genetic factors have been incriminated, such as genetic polymorphisms of the kidney and brain expressed protein (KIBRA; [93, 164]) and of the dopamine D2 receptor (DRD2; [139]) genes. In terms of biological factors and as mentioned in the Supplementary table, the cerebrovascular reserve, the integrity of noradrenergic system, and the initial size of the hippocampal volume, as well as vascular health, showed a significant effect on cognitive abilities during aging.

*Environmental factors* can also show significant effects on cognitive aging. Social interactions can have a significant role in improving mental health and general well-being [190]. In addition to physical interactions, virtual interactions (e.g. via social media) can help older people in dealing with stress and loneliness [105].

Overall, as mentioned in the column “Cognitively stimulating activities” (Supplementary table), education [3, 10], occupational status [142, 189], premorbid IQ estimation, and engagement in stimulating physical [140, 148], intellectual, and leisure activities [83, 178, 201], as well as a balanced diet and nutrition [54, 56, 57], might all have a neuroprotective role in aging. The wide concept of “activity” includes both social interactions with one or more people and more solitary activities such as reading or gardening [124]. Participation in leisure social activities seems to have a beneficial role on executive performance [151], and it is associated with a lower risk of developing dementia [197]. Regarding lexical production and aging, a recent study [83] found a positive correlation between the frequency of group activities and naming performance, as well as between social activities and left superior and medial frontal gyrus activation during picture naming. These results suggest that social leisure activities may contribute to maintaining the lexical production performance in older adults, through the neural and cognitive reserve mechanisms mainly dependent on the left prefrontal cortex. This region and particularly the superior medial frontal gyrus are involved in accessing semantic representations that can be guided by the emotional state. These results coincide with current studies showing a positive impact of emotional qualities of social activities [20] on global well-being and cognitive aging of older adults. We claim that regular practice of social leisure activities would modulate behavior, as well as brain activity, probably by boosting the affective drive and improving the efficiency of lexico-semantic search processes. Such activities require people to communicate more which results in more training. When people meet together, they are expected to talk and share ideas. Therefore, they can be expected to use language more than when they are not interacting with other people. This way, they regularly train their lexico-semantic search. In addition to positive effects related to emotion processes, the relation between the frequency of participation in social activity and language production scores may also be explained by preserved perspective-taking skills which might be beneficial. Perspective-taking refers to the ability to adopt the point of view of other people. It has been suggested that perspective-taking may be considered as a strategy to strengthen social bonds and is beneficial in several ways, for instance by favoring social coordination through increasing self–other overlap [62] which facilitates inter-human communication. Other explanations can be taken into account. According to Eyme et al. [52], a more active lifestyle in general is associated with increased gray matter volume in frontal areas associated to self-awareness and working memory. Additionally, many studies show a beneficial effect of social engagement on the protection against cognitive decline [14, 89, 173]. Specifically, social network size mediates the relation between an active lifestyle during middle adulthood and better cognitive functioning in old age [160]. In addition, a recent review showed that foreign language learning even at an advanced age has a positive impact on the maintenance of cognitive abilities, probably also *via* better socializing and integration into society, with a positive influence on their well-being [97]. However, the underlying factors that are responsible for the observed beneficial effects remain to be understood. Previous studies proposed stress prevention [60], reduction of depressive symptoms [37], forms of cognitive enrichment [79], and cognitive reserve [161]. In addition, emotional regulation processes could also play an important modulatory role between social activities and cognitive aging. Conversely, habits such as smoking or an inappropriate diet that can lead to biological dysfunctions can also have a deleterious effect on cognition during aging. Typically, high blood pressure and cholesterol, as well as diabetes, induce cognitive decline during aging (see [6, 7, 177]). The role of environmental factors, beneficial or deleterious, may be reflected in BDNF protein secretion which respectively stimulates or inhibits endogenous neurogenesis, especially during aging [65, 186].

*Bilingualism and education* are factors that showed modulation of various cognitive functions, even if the results are not consistent among authors. As mentioned in Supplementary Table and illustrated by the results of several studies that found such modulations, speaking two or several languages would improve executive and visuo-spatial functions and would delay the onset of neurodegenerative disorders of several years, by improving the cognitive reserve. Interestingly, early bilingualism would also be associated with lower CSF total-tau and lower prevalence of preclinical Alzheimer’s disease. In relation with the noradrenergic system mentioned previously, bilingualism would induce a sustained activation of this system, explaining the beneficial effects on cognitive functions. Moreover, next to cognitive reserve, bilingualism/multilingualism would also modify the cerebral reserve, typically the thickness of several white matter fascicles or even cortical regions, such as the anterior cingulate cortex. Overall, according to studies, daily bilingual experience mitigates the typical effects of aging on cognitive functions at both, behavioral and neural levels. As mentioned in Supplementary table, education has a significant role to maintain the cognitive level during aging. Indeed, working memory, several executive functions, and visuo-constructive abilities are modulated by education (see [150]) by modulating several cognitive reserves for these functions. Some other competencies (fluency, divided attention, interference, spatial reasoning) do not seem to be correlated to education.

As indicated in the same table (column “Physical activity and other factors”), other AMF seem to be closely related to cognition during aging, such as sleep and microbiota. Indeed, poor sleep would be associated with reduced cognitive flexibility and altered microbiome composition in older adults. Altered composition of the gut microbiome may be a mechanism linking inadequate sleep to low cognitive abilities in older adults. In addition, a cognitively enriched environment that may be achieved through education may be associated with right-lateralized fronto-parietal networks, which in turn contributes to the preservation of cognitive function in aging by offsetting the age-related decline in the ability to ignore salient distraction, as mentioned by Shalev et al. [169]. Indeed, as the authors explain, the ability to suppress distractors with age is driven by the right lateralization of neural substrates (including the fronto-parietal attention network, with a key role in cognitive reserve. Physical fitness and physical activity in general are factors frequently studied as having a positive effect on electrical brain activity and cognitive functions. Their influence is manifested either through direct modulation of executive functions, by reduced obesity or even by modulating the level of choline in the brain and allowing the synthesis of neurotransmitters, or by modulating the effect of APOE-ε4. An interesting 27-year longitudinal study [145] explored the effect of regular physical activity on cognition. The authors showed a significant beneficial effect on global cognitive function in older adults that would be gender-independent, not affected by differences in survival or by potential lifestyle confounders.

As mentioned previously, these factors do not act in isolation, but are rather interrelated. Papenberg et al. [136] suggest that the gene–environment correlation is particularly important and a specific genotype can be more frequently associated with a particular environment. This could explain why individuals with an advantageous genetic profile tend to seek for a more stimulating environment. In turn, a stimulating environment enhances the expression of particular genes. In line with this, Bartrés-Faz & Arenaza-Urquijo [12] hypothesized that the exposure to a protective and stimulating environment associated with a genetic predisposition can provide a high potential for adaptive neuroplasticity, in terms of reserve and neural compensation. A systematic review by Ngandu et al. [132] suggested that cognitive decline in seniors at risk of pathological aging can be avoided by a multi-domain intervention which involves diet, exercise, cognitive training, and vascular risk monitoring. The 2020 Lancet Commission report [110] completed a list of modifiable risk factors of pathological aging including excessive alcohol consumption, head injury, air pollution, less education, hypertension, hearing impairment, smoking, obesity, depression, physical inactivity, diabetes, and infrequent social contact. According to this report, about 40% of neurodegenerative disorders could be delayed or even prevented if a global care plan based on these factors was deployed in older adults while they are healthy. Finally, in a recent study, Belleville et al. [16] have shown in the StayFitLonger study that a multi-domain training that includes physical and cognitive activities may improve physical and cognitive health in older adults.

## LARA model: Lexical Access and Retrieval in successful Aging

**Fig. 1:**
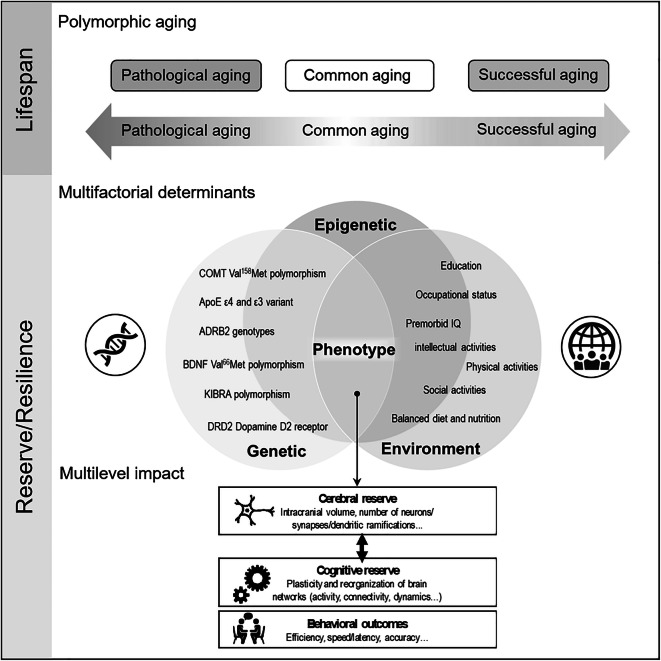
Complex mechanisms associated with aging. Aging is polymorphic and three general forms of aging are described: pathological/problematic, common/usual, and successful aging. Although classically considered to be categorical, these forms of aging can also be seen as a continuum (spectrum of aging; e.g., [59]). The underlying determinants of these different forms of aging are multifactorial and depend on various genetic, environmental, and epigenetic factors (see text for a description). Concerning the “gene–environment correlation” in aging, specific genotypes are more frequently associated with a particular environment and individuals with an advantageous genetic profile seek stimulating environments. Reciprocally, stimulating environments enhance the expression of favorable genetic profiles along with epigenetic mechanisms [136]. These different factors have an impact on the phenotypic profile of individuals and, as concerns the neurocognitive phenotype, at several levels of brain structure and function. More precisely, these modulating factors influence the capacities of neural (cerebral) and cognitive reserves (*aging-modulatory reserve factors*, AMF). These two forms of interrelated reserves are directly related to behavioral performances such as in lexical access and retrieval, particularly affected in aging

**Fig. 2:**
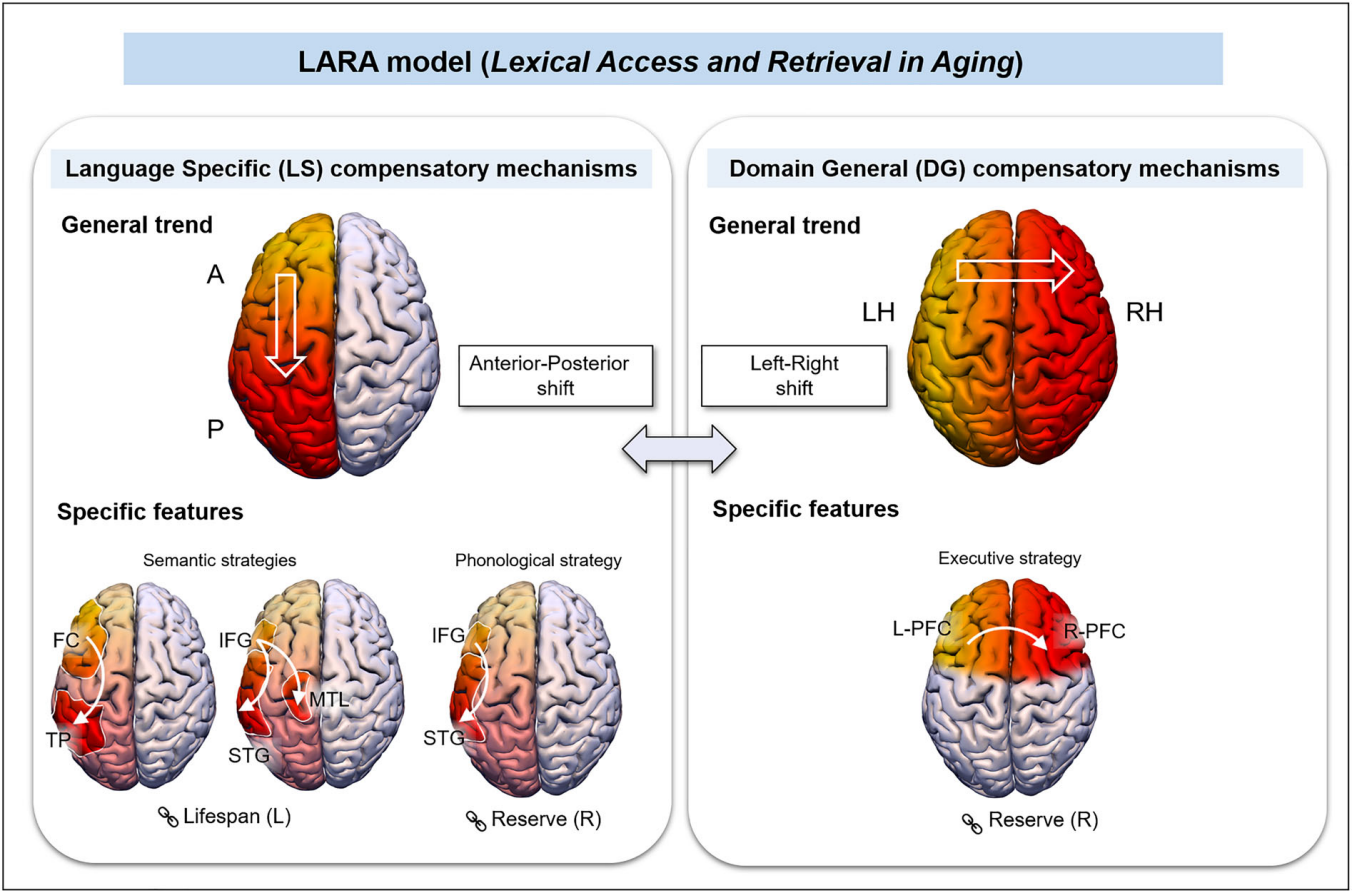
LARA model (*Lexical Access and Retrieval in Aging*). Neurocognitive compensatory strategies associated with successful Lexical Access and Retrieval in Aging. LARA model postulates the existence of two interrelated strategies: language-specific and domain-general (LS-DG). Language-specific mechanisms imply as a general trend a shift in recruitment from the frontal regions to posterior areas. Two main strategies are typically observed. (1) Semantic strategies that are related to universal aging (lifespan aspects) and implying an over-recruitment of temporo-parietal and mesial temporal regions. More specifically, this is characterized by (i) an increased lexico-semantic representation related to supplementary activation of temporo-parietal regions; (ii) an increased access to semantic memory knowledge store engaging top-down from inferior frontal medial temporal cortices; and (iii) an increased within-DMN connectivity with stronger access to conceptual and semantic representation. (2) A phonological strategy, dependent on cognitive reserve capacity, is characterized by (i) increased connectivity between the left inferior frontal and left superior temporal gyrus to overcome phonological deficits in aging. Domain-general mechanisms generally involve recruitment from the contralateral hemisphere (traditionally the right hemisphere in the case of language). This bilateral recruitment is observed in particular in the (pre)frontal regions (executive strategies) and will also depend on the cognitive reserve (see text for more details). *A*, anterior; *P*, posterior; *FC*, frontal cortex; *TP*, temporo-parietal regions; *IFG*, inferior frontal gyrus; *STG*, superior temporal gyrus; *MTL*, mesial temporal lobe; *LH*, left hemisphere; *RH*, right hemisphere; *L-PFC*, left prefrontal cortex; *R-PFC*, right prefrontal cortex. There are three main aspects of the LARA model. First of all, it considers two dynamics: (a) a universal or uniform one that refers to the general effect of age on cognition during *lifespan* and (b) an idiosyncratic or non-uniform one that refers to cognitive aging variability among individuals, depending on the amount of *reserve* or cognitive resilience of each individual. Secondly, these two dynamics are tightly and dynamically interrelated. They intervene in parallel and at any moment of aging and prompt the development of compensatory mechanisms. These mechanisms are implemented at a cognitive and cerebral level. Thirdly, LARA differentiates between two categories of compensatory mechanisms, language-specific and domain-general. To summarize these three accounts, LARA elements can be presented along two perspectives: (i) Lifespan vs. Reserve (L-R) mechanisms and (ii) Language-Specific vs. Domain-General (LS-DG) mechanisms, both types presenting interactions either within-side (L and R; LS and DG) or between-sides (L-R and LS-DG)

One way to synthesize and conceptualize how aging affects lexical production and cognition in general is through a neurocognitive model that integrates possible mechanisms underlying lexical difficulties in aging. We propose a comprehensive model, LARA, for *Lexical Access and Retrieval in Aging*. LARA describes the strategies and mechanisms gradually put in place during aging to perform lexical access and retrieval (see Figs. 1, 2 and Table 1).

An integrative view of compensatory mechanisms is also represented by the Scaffolding Theory of Aging and Cognition (STAC; [138]), based on compensatory scaffolding. According to STAC, additional networks are recruited in older adults, in interaction with defective or less-functioning ones, to maintain correct normal functioning. STAC assumes that a large cognitive reserve could determine the quality, quantity, and efficiency of compensatory scaffolding [138]. A revised version of the STAC model (STAC-r) was proposed [147] to incorporate the role of lifestyle factors that could increase or deplete brain resources and cognitive reserve. The LARA model is in line with these assumptions and our model can be seen as one of STAC-like models.

As concerns Lifespan and Reserve components (L-R), the mechanisms developed can be mostly L- or R-related, even if they may intersect. For instance, L-related strategies may be implemented to overcome increased latencies observed initially, related to the universal effect of aging during lexical production. They are mostly semantic strategies expressed either by (a) supplementary recruitment of lexico-semantic representations and increased activity of temporo-parietal regions [18] along the anterior (frontal)-posterior (lateral temporo-parietal) axis [82] or (b) a supplementary access to stored knowledge and semantic memory [26] triggered by top-down activation from frontal to medial temporal areas including hippocampi [84]. In line with this account, Catheline et al. [30] reported a significant correlation between task performance (verbal fluency) and hippocampal volume in older adults. The hippocampus has indeed an important role in semantic memory, allowing flexible maintaining and updating of semantic representations [47, 98]. On the other hand, R-related strategies can explain inter-individual variability in cognitive aging, since older adults are cognitively more or less successful depending on the amount of their reserve. For instance, during lexical production, lower-performing older adults, who display reduced accuracy and increased response latencies compared to younger adults, might not be able to rely only on semantic (L) strategies described above and additional (R) compensatory mechanisms need to be recruited. A compensatory mechanism for overcoming this difficulty could be achieved through a stronger connectivity between the left inferior frontal and left superior temporal gyri involved in phonological processing.

Therefore, we propose that in order to overcome lexical production difficulties, older adults universally implement semantic (L) strategies, and depending on their individual capacity to produce words, they might need to employ supplementary phonological (R) strategies.

The L-R compensatory strategies could be also described in terms of intrinsic TF (task-free) brain activity and various resting-state networks. This dimension, in interaction with TI (task-induced) activity, should also be considered in order to explain the cognitive evolution of adults as they age. As mentioned previously, the universal dimension of aging implies a specific pattern with reduced intra-network segregation and increased inter-network interaction, either between DMN network and executive-control and language networks [127] or between intrinsic networks such as DMN, FPN, and visual networks [77]. According to the authors, these changes occurring within and between intrinsic networks in interaction with extrinsic ones reflect age-related adaptive dedifferentiation or reduced specificity, and compensation mechanisms. They are in line with observations reporting increased between-network and reduced within-network connectivity [29, 31, 95]. Besides these general considerations on TF changes during aging, some of the results could be related to a specific strategy. For instance, in support of a semantic strategy, Krieger-Redwood et al. [100] showed increased intrinsic connectivity between the anterior temporal lobe and the medial prefrontal cortex, within the DMN. This increased within-DMN connectivity may reflect stronger access to conceptual and semantic representation, as accounted by the semantic strategy.

The main assumption within the language-specific–domain general (LS-DG) perspective is that word production difficulties observed in the elderly involve mechanisms and compensatory strategies which include both language-specific and domain-general processes, mostly executive functions depending on prefrontal cortices. As reported above, a large panel of TI studies [21, 125, 146] and TF studies [5, 38, 50, 69, 154, 202, 209] revealed various reorganization patterns at a prefrontal level, with a tendency toward reduction of hemispheric asymmetry. As we mentioned above, naming latencies in older adults were modulated by the degree of inter-hemispheric asymmetry of frontal regions, with shorter naming latencies being correlated with more bilateral activity in frontal regions, revealing a compensatory mechanism via executive functions, a result that is also supported by the HAROLD model [28]. These findings suggest that domain-general executive strategies are also recruited in older adults, to help maintain processing speed and improve reaction times. Specifically, left frontal cortices might be involved in translating affective drive states into a coordinated plan to help retrieve semantic information from memory [17].

In the column “LARA-based mechanisms” from Table 1, we interpreted the results of studies we synthesized from the last 20 years on lexical production and aging, according to mechanisms possibly involved according to our proposed LARA model. Among the 46 studies cited in Table 1, all except one can be interpreted according to LARA mechanisms. Results of the majority of studies are in line with language-specific mechanisms only (20/46) while a few (5/46) with domain-general mechanisms. The majority of studies (21/46) reported results that could be in line with both, domain-general and language-specific mechanisms. Furthermore, more than a half (25/46) of studies can be interpreted according to both lifespan and reserve mechanisms. Overall, our interpretation within the framework of LARA mechanisms shows that cognitive aging of lexical production is a multidimensional phenomenon that should be explored along at least two dimensions, lifespan and idiosyncratic. Even if our approach is only semi-quantitative and our interpretation may be more or less subjective, results reported by these studies show a large variety of possible mechanisms explaining the ability of each individual to cope with aging effects, according to his/her own life trajectory and his/her level of cerebral, neural, and cognitive reserve. We also mentioned in Supplementary Table the main AMF that modulate the cognitive and cerebral reserve and claim that they are multidimensional and their effects are interconnected. It is difficult to disentangle, in the same individual, the effect of this or that specific factor. We rather propose that for each individual, we can depict a composite reserve index representing a group of interrelated factors, some of them being beneficial and some others deleterious for the cognitive evolution while aging. This composite reserve index clearly depends on the specific life and health trajectory of an individual, its education in a grad sense, style, and quality of life.

## Conclusions

Cognitive aging is an inevitable process which affects all individuals. An important amount of studies has been conducted to assess the effect of age on cognitive functioning and to determine cognitive, behavioral, and anatomo-functional correlates and biomarkers of this process. A specific cognitive function addressed in this review was lexical production, which is generally impaired in normal aging. We showed that the decline in lexical production skills is variable among older adults, according to the variable amount of reserve. Older adults implement a variety of compensatory strategies to maintain lexical performance as long as possible and avoid pathological aging. The LARA model provides an overview of compensatory strategies and the way in which they are implemented in older adults, in terms of cognitive mechanisms and cerebral networks. These strategies relate both to language-specific mechanisms and to domain-general, executive functions. We argue that the degree and variability of cognitive aging depend on the amount of reserve specific to each individual and that they are modulated by a large panel of AMF factors. A variety of mechanisms and compensatory strategies coexist in the same individual to compensate for complex deleterious effects of aging, LARA describing only some of them. A better understanding of these strategies and of the protective and risk factors for pathological aging is critical to society and public policies, to promote successful aging and avoid or push back the occurrence of neurodegenerative disorders.

In conclusion and as mentioned in Sholl and Rattant [163], aging is one of the most challenging public health issues, considered as a “cellular danger response to environmental stressors or injury leading to the development of neurodegenerative disorders.” New research directions have recently emerged to understand the effect of genetic, biological, social, and environmental factors on cognitive reserve in each individual. The role of life conditions, environment, physical activities, or nutrition seems to be also significantly considered. Identifying personalized biomarkers of the cognitive reserve can help characterizing biologically vulnerable individuals and the implementation of strategies that may help neurobiological changes at a cerebral level, reflected for instance, by increased brain connectivity and new circuits and strategies to overcome cognitive weaknesses. Overall, these new research directions will help elucidating the role of beneficial AMF factors in promoting a successful aging.

## Supplementary information

ESM 1 Synthetic presentation of main studies reporting results on modulatory factors of the cognitive reserve during aging in healthy older adults. All information on the inclusion of studies are presented in the main text. (DOCX 34 kb)
