# Supplementary material for: Strategies and cognitive reserve to preserve lexical production in aging
Source: GeroScience. 2021 May 10;43(4):1725–65. doi: 10.1007/s11357-021-00367-5 (PMC8492841; doi:10.1007/s11357-021-00367-5)
Supplement: Supplementary file 1 — Synthetic presentation of main studies reporting results on modulatory factors of the cognitive reserve during aging in healthy older adults. All information on the inclusion of studies are presented in the main text. (DOCX 34 kb) [file 11357_2021_367_MOESM1_ESM.docx]

| **Genetic** | **Biological** | **Cognitively stimulating activities** | **Bilingualism & Education** | **Physical activity & Other factors** |
| --- | --- | --- | --- | --- |
| **Tupler et al., 2007**  <https://pubmed.ncbi.nlm.nih.gov/16916565/>  **APOE-4:** predicts longitudinal memory decline in healthy aging. | **Tupler et al., 2007**  <https://pubmed.ncbi.nlm.nih.gov/16916565/>  **Hippocampal volume:** MRI morphometry of the hippocampus adds to the predictive value of APOE-4. | **Wilson et al., 2003**  <https://pubmed.ncbi.nlm.nih.gov/12815501/>  **Cognitively stimulating activities:**  Lifelong frequency of participation in cognitively stimulating activities is associated to cognitive functioning in older adults. More frequent cognitive activity was related to better perceptual speed, visuospatial ability, and semantic memory. | **Gollan & Ferreira, 2009**  <https://pubmed.ncbi.nlm.nih.gov/19379041/>  **Bilingualism and voluntary switch between languages.** The freedom to mix languages voluntarily allows unbalanced and older bilinguals to function more like balanced and younger bilinguals. Voluntary switch costs reveal an expanded role for inhibitory control in bilingual language production and imply a mandatory separation by language in bilingual lexical selection. | **Fitzpatrick et al., 2007**  <https://pubmed.ncbi.nlm.nih.gov/18000144/>  **Physical activity (fast-paced walking):** predicted early cognitive. decline. Slower walking speed identified in older adults with lower cognitive functioning may reflect very early physical changes that precede neurological/psychological symptoms of dementia. |
|  |  | **Fritsch et al., 2007**  <https://pubmed.ncbi.nlm.nih.gov/17565095/>  **Mental activities, educational pursuits, engaged lifestyle in adolescent period:** IQ in adolescents has strong effect on global cognitive functioning in aging (mainly episodic). This is also related to high school mental activities and education. Mental activities have direct effects on verbal fluency in old age, but physical and social activities did not predict cognitive functioning in aging. Education show direct effects on global cognitive functioning in later life, specifically on episodic memory, and processing speed. Midlife factors such as occupational demands did not predict late-life cognition. Cognitive reserve is dynamic and most amenable to change in early life. An active, engaged lifestyle, emphasizing mental activity and educational pursuits in early life, can act as a protective factor on cognitive functioning in later life. |  | **Eskes et al., 2010**  <https://pubmed.ncbi.nlm.nih.gov/21048898/>  **Physical fitness:** Physical fitness predicts global cognitive functioning, specifically for attention and executive functioning older women. |
| **Van Gerven et al., 2012**  <https://pubmed.ncbi.nlm.nih.gov/22642392/>  **APOE-ε4:** there was no evidence that older, high-educated carriers decline cognitively more than younger, as well as older low-educated and noncarriers. | **Eskes et al., 2010**  <https://pubmed.ncbi.nlm.nih.gov/21048898/>  **Cerebrovascular reserve:** predicts the global cognitive functioning, specifically for attention and executive functioning older women. | **Slegers et al., 2009**  <https://pubmed.ncbi.nlm.nih.gov/19225269/>  **Computer training:** Intensive interaction with a personal computer with standard software applications has no effect on cognitive measures in older adults. The results of this study do not provide support for the notion that stimulating the cognitive skills of older increases their cognitive abilities. | **Kousaie et al., 2014**  <https://pubmed.ncbi.nlm.nih.gov/25120442/>  **Bilingualism :** The results suggest that there may be an influence of the language environment. It is concluded that additional research is required to fully characterize any language group differences in both executive function and language tasks. | **Kirton et al., 2016**  <https://pubmed.ncbi.nlm.nih.gov/26667889/>  **Obesity:** Cognitive reserve (years of education) protects against BMI (body mass index)-related executive dysfunction in younger adults, but not in older adults. The cumulative effect of age-related cognitive decline and increased body weight may minimize any protective benefit of cognitive reserve on executive functioning. |
| **Pool et al. 2016**  <https://pubmed.ncbi.nlm.nih.gov/26984944/>  **APOE-ε4:** greater occupational cognitive demands are associated to significantly better late-life and slower decline in global cognitive functioning.  Faster annual decline in carriers APOE e4 genotype, but the association of occupational cognitive requirements and the rate of cognitive decline did not differ significantly by APOE e4 carriership.  Adulthood cognitive activity can contribute to cognitive reserve in late life. | **Clewett et al., 2016**  <https://pubmed.ncbi.nlm.nih.gov/26521135/>  **Integrity of the noradrenergic system:** Older adults with low cognitive reserve rely more in normal noradrenergic system function to promote or maintain executive function. Locus coeruleus (LC) neuromelanin signal intensity is associated with cognitive reserve especially with verbal intelligence. In older who are vulnerable to cognitive decline, the integrity of the noradrenergic system helps supporting cognitive flexibility. Intellectually engaging experiences protect cognitive health in later adulthood. | **Eskes et al., 2010**  <https://pubmed.ncbi.nlm.nih.gov/21048898/>  **Cognitive stimulation activities:** a diversity of cognitive activities predicts global cognitive functioning, specifically for attention and executive functioning in older women. The effects appear to be more related to the diversity of activities rather than their duration. | **Calvo et al., 2016**  <https://pubmed.ncbi.nlm.nih.gov/26793100/>  **Bilingualism and cognitive reserve (CR) :** beneficial effect on CR, would delay the onset of AD. Authors say that it is important to explore not just the delay of AD in bilinguals, but the changes occurring throughout the course of disease. These considerations could help us tease apart the potential contributions of bilingualism to preserved functioning across cognitive domains. Important to explore the relationship between bilingualism and CR, but also on general mechanisms of cognitive compensation. | **Matura et al., 2017**  <https://pubmed.ncbi.nlm.nih.gov/28934191/>  **Aerobic exercise training:** Cerebral choline concentration remains stable in healthy older that had 12 weeks aerobic exercise while the concentration increased in the control group. The stable choline concentration in the intervention group over 3-month suggest a neuroprotective effect of aerobic exercise in aging adults. *(NB. the choline allows the synthesis of neurotransmitters in the brain, in particular acetylcholine, beneficial for cognition).* |
| **Lopez et al., 2017**  <https://pubmed.ncbi.nlm.nih.gov/29414814/>  **APOE-ε4:** carriers show poorer execution in several cognitive domains including global cognitive functioning, episodic memory, verbal fluency, and naming, more noticeable in older and less educated subjects. APOE4 genotype influences cognition in aging in interaction with other factors. | **Fischer et al., 2014**  <https://pubmed.ncbi.nlm.nih.gov/24465994/>  **Network compensation capability:** In advanced aged there is association with alterations of network properties and general intelligence. Network alterations that occur in aging may only have an effect on general intelligence once the characteristics of the network have deteriorated such that efficient communication and integrated processing between grey matter regions is impaired. The individual ”network compensation capability” might be a surrogate of cognitive reserve or brain resilience in older adults. | **Saint Martin et al., 2017**  <https://pubmed.ncbi.nlm.nih.gov/29100612/>  **Social engagement:** High cognitive abilities and social involvement are related. Protective role of cognitive reserve reflected inone-step decline in the “cognitively elite” and “cognitively normal” adults that extended the time to reach an impaired level of cognitive function. Strong correlation between staying socially engaged later in life and stability of cognitive functioning. | **Roldan-Tapia et al., 2017**  <https://pubmed.ncbi.nlm.nih.gov/29118710/>  **Education:** Performance in all cognitive domains decreased with age, effect attenuated by education, mainly in visuo-constructive domain. Visual and verbal memory tests are not affected either by aging, education, or cognitive reserve. Cognitive domains such as working memory, several executive functions and the visuo-constructive abilities are modulated by education. The limited effect of cognitive reserve (education) on fluency, divided attention, interference, spatial reasoning, and visuospatial tasks could be due to the brain’s own compensatory mechanism, independent of education or cognitive reserve in older adults. | **Lopez et al., 2017**  <https://pubmed.ncbi.nlm.nih.gov/29414814/>  **Education:** Educational seems to modulate the effect of APOE-ε4. |
| **Ward et al., 2017**  <https://pubmed.ncbi.nlm.nih.gov/29067339/>  **BDNF Val66Met polymorphism:** moderated the relationship between cognitive reserve and change in executive functions. Expected positive association of lifetime exposure to cognitively stimulating activities and cognitive performance, is weaker in BDNF Met than Val carriers. | **Baker et al., 2017**  <https://pubmed.ncbi.nlm.nih.gov/26961092/>  **Length of white matter tracts:** Reduced cognitive functioning in aging is associated with shorter white fibers length (FBL) and lower cognitive reserve (estimated by premorbid IQ). Cognitive reserve moderates the relationship between FBL and RBANS. Lower cognitive performance is observed in older adults with low cognitive reserve and short FBL. In older with high cognitive reserve, the relationship between FBL and cognitive performance is reduced. Cognitive reserve serves as protective factor in older adults against lower cognitive performance and despite age-associated reductions in FBL. | **Clare et al., 2017**  <https://pubmed.ncbi.nlm.nih.gov/28323829/>  **Social interaction and social isolation:** Isolation in later life is detrimental to cognitive health. Cognitive reserve moderates association between social isolation and cognition at 2-year follow-up.  Maintaining a socially active lifestyle in later life enhance cognitive reserve and benefit cognitive function. Negative effect of social isolation is based on receiving less cognitive stimulation through social contact, resulting in lower cognitive reserve and poorer cognitive function. | **Estanga et al., 2017**  <https://pubmed.ncbi.nlm.nih.gov/27916386/>  **Bilingualism and CSF biomarkers.** Early bilingualism was associated with lower CSF total-tau and lower prevalence of preclinical AD. Bilingualism showed a moderation effect on the relationship between age and CSF AD-biomarkers and the relationship between age and executive function. Bilingualism contributes to cognitive reserve enhancing executive and visual-spatial functions. | **Ji et al., 2018**  <https://pubmed.ncbi.nlm.nih.gov/29851152/>  **Dance training:** Six‐week exercise training (dancing) improved gait speed, cognitive function and compensatory ability through increased involvement of motor‐related networks in older adults.  Older adults who activated motor network only after exercise (but not before exercise) showed better logical memory performance. This robust neural compensatory mechanism can be considered as part of cognitive reserve. |
| **Snitz et al., 2020**  <https://pubmed.ncbi.nlm.nih.gov/32699143/>  **APOE*2** is protective against Aβ deposition, and associated with better initial cognitive performance, paid work engagement and life satisfaction. | **O’Shea et al., 2018**  <https://pubmed.ncbi.nlm.nih.gov/30467475/>  **Hippocampal volume:** positive main effect between larger hippocampal volume and memory performance is strongest in those with high level of education. Higher level of education or cognitive reserve, enable older adults to optimize structural integrity of the hippocampus to support delayed recall. | **Evans et al., 2018**  <https://pubmed.ncbi.nlm.nih.gov/30118489/>  **Social interaction:** Being socially integrated in later life is beneficial to cognition. Cognitive reserve moderates the association of social isolation and cognition, reflecting the importance of being engaged throughout the lifespan in order to build reserve to protect against poor cognitive function in later life.  Good social interactions may be more beneficial to cognition in individuals with low mid-life reserve, as measured by occupational complexity. | **Bak & Robertson, 2017**  <https://pubmed.ncbi.nlm.nih.gov/27866671/>  **Bilingualism, cognition.** Sustained activation of noradrenergic signaling pathways associated with bilingualism could provide a possible mechanism linking explaining the delayed onset of dementia in bilinguals. | **Sanchez-Lopez et al., 2018**  <https://pubmed.ncbi.nlm.nih.gov/29370215/>  **Physical activity:** Significant difference observed in cognitive functioning of older with respect to physical activity. The active older adults had higher IQ and better performance for processing speed, matrix reasoning, digit-symbol coding, and picture arrangement. Active older adults showed less EEG delta and theta activity, and more alpha activity than the passive group, mainly in frontotemporal areas. Higher levels of physical activity are positively associated with cognition and brain electrical activity. |
|  | **Harrison et al. 2018**  <https://pubmed.ncbi.nlm.nih.gov/29665578/>  **Hippocampal volume:** Older people with initially high memory levels, show lower decline than those with lower levels. Hippocampal volume and regional cortical thickness show greater values in successful aging olders, while they had similar levels of brain amyloid compared to typical older. Successful aging trajectory reflected in high memory level is supported by a combination of several protective factors: brain reserve, resistance and resilience to pathology (Aβ accumulation). | **Piccirilli et al., 2019**  <https://pubmed.ncbi.nlm.nih.gov/31543727/>  **Social (discussion participation, travelling in group) and mental activities (listening audio tracks with various topics):** Older adults that had multimodal trainings during six months significantly improved their cognitive performance including attention, processing speed, memory, and executive functions, as well as mood state, in comparison to their pre-test levels and the control elderly subjects. An active lifestyle protects against cognitive decline with age. | **Anderson et al., 2018**  <https://www.ncbi.nlm.nih.gov/pmc/articles/PMC5845836/>  **Bilingualism and white matter (language):**  Authors used DTI to compare monolinguals and bilinguals; show that bilinguals still had greater axial diffusivity (AD) in the left superior longitudinal fasciculus supporting a neural reserve account for healthy older bilinguals. | **Engeroff et al., 2018**  <https://pubmed.ncbi.nlm.nih.gov/30077618/>  **Physical activity:** significant association between brain plasticity outcomes and physical activity but not with performance. BDNF was detrimentally associated with sedentary-time but beneficially related to accelerometer total activity counts and moderate to vigorous physical activity.  Regular physical activity (regardless of physical performance) is beneficial for preserving brain plasticity in aging adults, whereas sedentary behavior can have negative influence. |
|  | **Snitz et al., 2020**  <https://pubmed.ncbi.nlm.nih.gov/32699143/>  **Vascular health - pulse pressure:** APOE*2 and lower pulse pressure predict resistance to Aβ deposition in advanced aging (14 years prior, as premorbid abilities and predict cognitive status in  the presence of Aβ). Baseline pulse pressure predicts longitudinal Aβ increase. | **Park et al., 2019**  <https://pubmed.ncbi.nlm.nih.gov/30350714/>  **Handicraft work (e.g. gardening, knitting, DIY-*Do It Yourself*), social activities, voluntary work:** Education positively associated with cognitive function and craft activities positively predicted cognitive function in older. Education moderates relationship between leisure activities and cognitive function in aging adults.  Low-educated older adults showed a decrease in cognitive function as they performed domestic chores and an increase in cognitive function as they participated in social activities and volunteering.  High-educated elderly showed no relation between leisure activities and cognitive function. | **Borsa et al., 2018**  <https://pubmed.ncbi.nlm.nih.gov/29360516/>  **Bilingualism, cognitive and cerebral reserve.** VBM study showing cumulative effects of age on grey matter volume (GMV) in structures that are involved in cognitive control in bilinguals. Found that chronological age predicts the size of interference and conflict effects for monolingual speakers only; observed a more widespread pattern of bilateral aging-effects in regions classically associated with aging in monolingual speakers compared to bilingual speakers. GMV in the dorsal anterior cingulate cortex and the level of daily exposure to a second language independently predict performance on the attentional network in bilinguals. Daily bilingual experience mitigates typical effects of aging on cognitive control. | **Anderson et al., 2018**  <https://pubmed.ncbi.nlm.nih.gov/29031742/>  **Microbiota and sleep:** Poorer sleep results in reduced poor cognitive flexibility and altered microbiome composition in older adults. Altered composition of gut microbiome may be a possible mechanism linking inadequate sleep to reduced cognitive abilities in older adults. Improving microbiome health may buffer against sleep-related cognitive decline in older adults. |
|  |  | **Chen et al., 2019**  <https://pubmed.ncbi.nlm.nih.gov/31315089/>  **Intellectual and leisure social type activities:** Highly educated older with better cognitive functioning in multi-domains participated more in knowledge-related leisure activities and had slower age-related reductions of executive function. Intellectual and social type of leisure activities mediated the association between education and multiple cognitive domains (memory, language, attention and executive functions). There is a significant effect of age and education interaction on gray matter volume of the anterior brain regions and white matter integrity. The interaction between age and education affects cognition indirectly through the white matter integrity. Results suggest that high education in early life help to postpone the decline of cognitive and brain reserve in normal aging. |  | **Piccirilli et al., 2019**  <https://pubmed.ncbi.nlm.nih.gov/31543727/>  **Physical activity (walking):** Older adults that had multimodal training including walking during six months significantly improved their cognitive performance including attention, processing speed, memory, and executive functions, as well as mood state, in comparison to their pre-test levels and the control elderly subjects. An active lifestyle protects against cognitive decline with age. |
|  |  | **Mohammad et al. 2019**  <https://pubmed.ncbi.nlm.nih.gov/31656218/>  **Regular mental activities (reading, writing, calculation, crosswords and mind teasers, watching documentary, web searching, and social networking):** Older adults with distributed type of educational training had better memory performance than those with continuous educational training. There was no difference between bilingual and monolingual older adults. Mental activities such as reading, writing, doing calculation, playing crosswords and mind teasers, watching documentary, web searching, and social networking have a positive association with episodic memory.  Age, educational level and mental activities (playing mind teasers) predict episodic memory functioning. |  | **Reas et al., 2019**  <https://pubmed.ncbi.nlm.nih.gov/31356211/>  **Regular physical activity:** a 27-years longitudinal study found that concurrent physical activity has a significant beneficial age-dependent association with global cognitive function, executive function, memory, and verbal fluency. The associations of concurrent physical activity and cognitive function become more apparent with advancing age. These associations are gender-independent, not affected by differences in survival, and not affected by potential health and lifestyle confounders. Physical activity in early adulthood, but not during teenage years, increases the positive association between cognitive function and physical activity in aging adults. |
|  |  | **Snitz et al., 2020**  <https://pubmed.ncbi.nlm.nih.gov/32699143/>  **Paid work engagement** predicts less cognitive decline. | **Voits et al., 2020**  <https://pubmed.ncbi.nlm.nih.gov/32882240/>  **Bilingualism and brain reserve.** Bilingualism contributes to brain reserve in aging and show delayed onset of AD symptoms. Other links at the level of brain and behavior can be observed between bilingualism and clinical neurodegeneration that should be clarified. | **Snitz et al., 2020**  <https://pubmed.ncbi.nlm.nih.gov/32699143/>  **Life satisfaction** predicts less cognitive decline. |
|  |  | **Altieri et al., 2020**  <https://pubmed.ncbi.nlm.nih.gov/31495773/>  **Cognitive stimulating activities:** apathy is associated with lower cognitive reserve in all age groups; high level of cognitive reserve is associates with low level of apathy. Cognitive stimulating activities associated with lower levels of apathy at any age. No association between depression and cognitive reserve. |  | **Orland et al., 2020**  <https://pubmed.ncbi.nlm.nih.gov/32557334/>  **Physical fitness:** is a strong mediator of the relationship between age and processing speed.  Physical fitness has a major contribution to cognitive reserve in older. The decrease in physical fitness during aging may partially account for slower cognitive processing. |
|  |  | **Rodriguez et al., 2020**  <https://pubmed.ncbi.nlm.nih.gov/32819031/>  **Intellectual engagement, high demanding jobs in fields:** **“Language & Knowledge”, “Pattern detection”, “Information processing”, and “Service”:** Older adults who worked in jobs with higher demands throughout their life show slower cognitive decline later in life. High level demands in services mentioned above seem to have the strongest protective affects. APOE e4-allele carriers had in these categories. Intellectual engagement throughout the occupational history seems to protect cognitive functioning in old age even in APOE e4 allele carriers. |  | **Shalev et al., 2020**  <https://academic.oup.com/cercorcomms/article/1/1/tgaa049/5894593>  **Rich environment and right-lateralized networks:** Cognitively enriched environments, achieved through education, modify structural organization with right-lateralized fronto-parietal regions, which contributes to the preservation of cognitive function in aging by offsetting the age-related decline in ability to ignore salient distraction. The variability in the capacity to suppress distractors in older adults is driven by the right lateralized neural substrates of brain reserve, encompassing regions within the right frontoparietal attention network. Right lateralized network have a key role in cognitive reserve. |
|  |  | **Hassing et al., 2020**  <https://pubmed.ncbi.nlm.nih.gov/29304225/>  **Self-improvement activities (for men), intellectual–cultural activity (for women):** for older men, only self-improvement activity was related to higher levels of verbal, spatial abilities and speed. Higher domestic activity is related to less decline in speed processing. For older women, higher intellectual–cultural activity was related to higher level of verbal ability and memory and to steeper decline in memory while higher domestic activity was significantly associated with steeper decline in spatial ability and memory. Cognitively stimulating activities increase cognitive reserve. |  |  |
|  |  | **Ferré et al., 2020**  https://pubmed.ncbi.nlm.nih.gov/33250759/  **Functional Connectivity of Successful Picture-Naming:** **Age-Specific Organization and the Effect of Engaging in Stimulating Activities:** naming accuracy depends on posterior cingulate cortex (PCC) functional decoupling in both younger and older adults but through different pathways according to their performance. PCC decoupling mediates the relationship between the level of engagement in stimulating activities and naming accuracy in younger adults, but not in older adults. These findings suggest that reserve-related mechanisms may be more critical for naming in early adult life, while older adults' neurofunctional organization may benefit more from a lifetime of acquired knowledge. |  |  |
